# The fault systems that control the occurrences of iron ore deposits in northeastern Aswan, Egypt

**DOI:** 10.1038/s41598-025-88831-6

**Published:** 2025-02-06

**Authors:** Mohamed Abd El-Wahed, Ibrahim A. Salem, Mohamed Attia, Dina Younis

**Affiliations:** 1 Geology Department, Faculty of Science, Tanta University, Tanta, 31527 Egypt; 2 Geology Department, Faculty of Science, Kafr El Sheikh University, Kafr El Sheikh, 33511 Egypt

**Keywords:** Iron ore deposits, NE Aswan, Abu Subeira, Abu Aggag, Multi-sensor remote sensing data, Structural analysis, Environmental sciences, Solid Earth sciences

## Abstract

The present study combines remote sensing data (Landsat-8 and ASTER) with structural analysis to identify the fault systems affecting the distribution of the ironstone beds in northeastern Aswan, Egypt. Sedimentary rocks, such as the Abu Aggag, Timsah, and Umm Brammily formations, characterize northeastern Aswan. The Abu Aggag Formation consists of kaolinitic conglomerate, conglomeratic sandstone, and mudstone. The upper Timsah Formation consists of ferruginous sandstones, oolitic ironstone, and mudstone. Fluvial sandstone forms the Umm Brammily Formation. The oolitic sandy ironstone (2–2.5 m thick) is rich in dark red oolitic hematite and goethite. The ironstone deposit ranges in composition from oolitic sandy ironstone to oolitic ironstone. The distribution of iron minerals is extremely consistent with the iron concentration grade indicated by the Landsat-8 and ASTER Brand Ratios. Five main fault sets to control the extension of the ironstone beds: NNE-SSW left-lateral strike-slip faults (set 1); ENE-WSW normal faults (set 2); NNW-SSE normal faults (set 3); NW–SE normal faults (set 4); and NE-SW normal faults (set 5). The subsequent displacement of Set 3 faults increased the depth of the ironstone bed, which raised the sedimentary overburden load and increased the cost of ironstone exploitation, as well as the absence of iron ore exploitation zones bordering the Nile River.

## Introduction

**Fig. 1:**
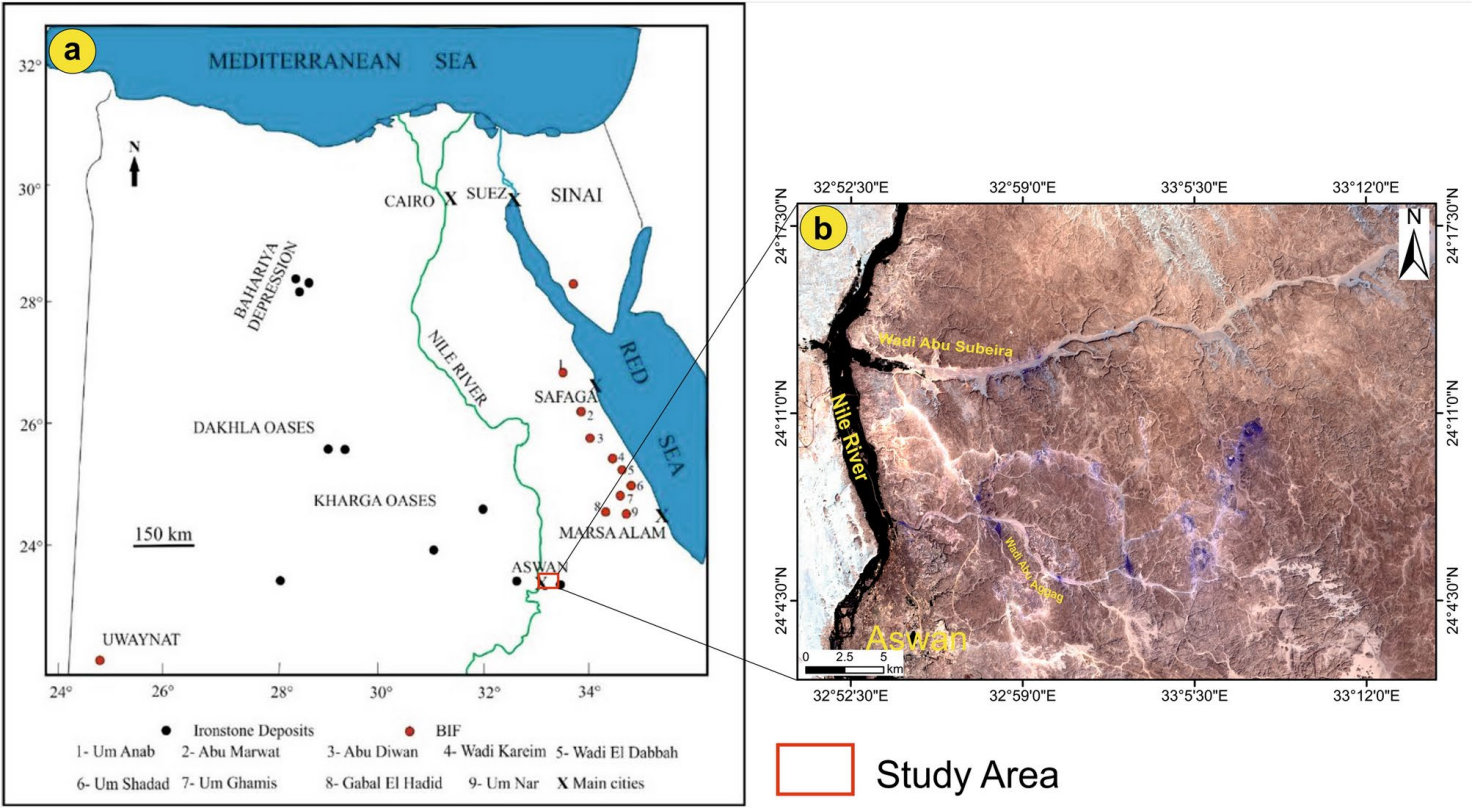
A location map of the study area (red box) over the Egyptian territory map showing (**a**) distribution of the important sites of the iron ore deposits (after^84^); (**b**) A color composite 234-RGB of Landsat-8 shows the outlines of the study area. (**b**) is a Landsat 8 image downloaded through USGS. The figure was created and processed by ENVI v. 5.6.2. software: https://www.l3harrisgeospatial.com/Software-Technology/ENVI), which is mainly utilized for image processing).

Iron ore is a vital resource for global economic development. Iron accounts for around 95% of all metals consumed each year, with China being the world’s largest user of iron ore and manufacturer of steel, followed by Japan and Korea^1–4^. In Egypt, iron ore deposits are represented in the Sinai Peninsula, Eastern Desert, Western Desert, and along the Nile Valley. According to the Egyptian Mineral Resource Authority^5^, nine main iron ore deposits may be found in Egypt, including those in the Aswan, Bahariya, Abu Marwat, Umm Ghamis El-Zarga, Um-Nar, El Oweinat, Gabal El-Hadid, Wadi Karim, and Wadi El-Dabbah regions (Fig. 1a). Recently, iron, ilmenite, and gold have been considered the main pillars of the Egyptian mining industry for metallic ores, as well as manganese and chromite (with small-scale mining). According to the tectonic–magmatic stages, Botros and Noor^6^ divided the Egyptian mineral deposits into the following categories: the island arc stage, the accretional stage (Orogenic stage), and the late orogenic-extensional stage, in which the Egyptian iron ores are represented by ironstone and the banded iron formation (BIF)^7^.

Ironstone in Egypt is well represented within the Phanerozoic sediments of Bahariya and Aswan^7^. It served as the primary source for the Egyptian iron and steel industry until Bahariya iron ore took its place. Hussein^8^ asserts that the Egyptian ironstone is a bedded oolitic type of the Senonian age that originated in sedimentary lacustrine settings. The ironstone is exposed as two bands, varying in thickness from 0.2 to 3.5 m, interbedded with ferruginous Nubian sandstone and clay. Hematite is considered the main iron mineral, with minor representations of magnetite, limonite, and goethite, accompanied by some gangue minerals (e.g., pyrite, siderite, quartz, gypsum, halite, and clay)^9^. Salem and El Gammal^10^ pointed out that these iron oxides are mainly represented in the western and northeastern parts of Aswan Lake, particularly along Wadi Abu Subeira, Wadi Abu Aggag, Um Baramil, Um Hebal, and Um Hakban, where the iron ore deposits are concentrated.

Remote sensing techniques have recently gained importance in mineral exploration due to their ability to save time and effort compared to manual field surveys, as well as the availability of data^13–16^. Consequently, the global search for iron ores has employed several spectral remote sensing techniques^10,16–22^. For instance, the detection of iron content was performed using Landsat 8 and ASTER data using various methods, including False color composite (FCC), Band Ratios (BRs), Principal Component Analysis (PCA), and Minimum Noise Fraction (MNF)^23,24^. When compared to Landsat OLI, the detailed spectral characteristics of ASTER data commonly lead to the mapping of probable iron ore zones^25–29^. We applied several spectral methods, such as Spectral Angle Mapper (SAM), Constrained Energy Minimization, and Mixture Tuned Matched Filtering, to the ASTER dataset to detect and map the ore deposits and hydrothermal alteration minerals^21,23,27,30–33^.

Several regular faults in diverse directions impact the iron ore in northeastern Aswan, the displacement of which has led to the formation of graben and horst, as well as the displacement of iron-rich zones. As a result, displacement along these faults controls access to the iron-bearing layer, as well as the cost of extraction. One of the most significant goals of this research is to investigate the fault orientations that generate these displacements. The presented study aims to detect potential iron ore zones by mapping the distribution of different iron oxide minerals using Landsat-8 and ASTER images. Additionally, the potential zones of iron content were linked and interpreted through a detailed structural analysis via Sentinel 1 data, field investigation, and structural measurements to manifest the effect of structural features in controlling iron ore deposits in the area under consideration, besides resolving the complicated structural features through introducing a structural/tectonic model for the investigated area.

## Study area and geological setting

**Fig. 2:**
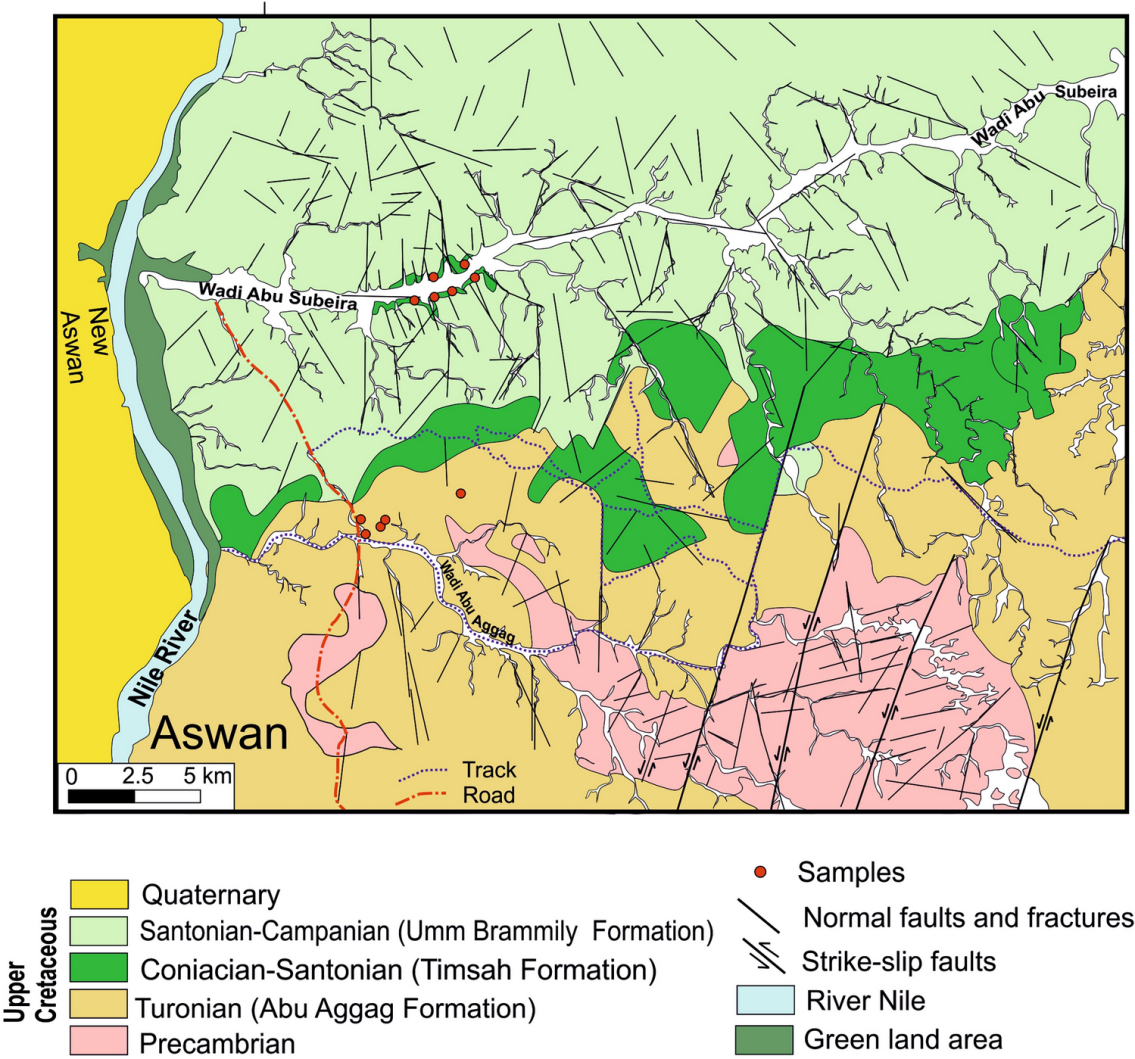
Geological map of the NE Aswan (modified after Klitzsch et al. 1987). (Created by SmartSketch v. 4.0 software; https://smartsketch.software.informer.com/4.0/).

**Fig. 3:**
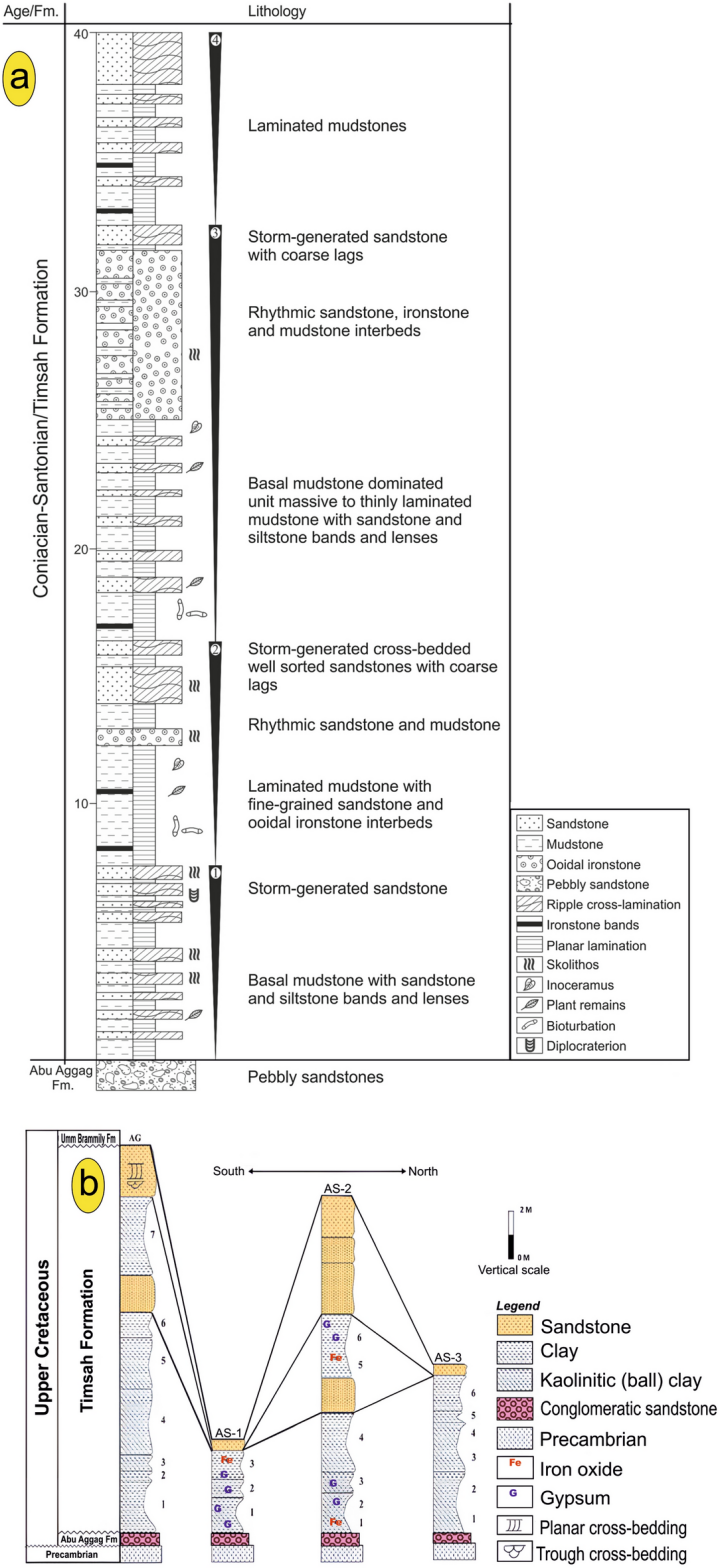
(**a**) A representation of the Timsah Formation’s upward cycles of shoaling and the location of the associated ironstones in the Gabal Timsah type locality in the southern region of Aswan, Egypt, modified after El Aref et al.'s (1996) and Salama’s (2014) work. (**b**) An overview of the lithostratigraphic columnar sections of Abu Aggag (AG) and Abu Subeira (AS-1, 2, and 3) located in the northeast region of Aswan, Egypt, as illustrated in Sharaka et al.'s (2022) work.

The investigated area is located in the northeastern region of Aswan City, situated to the east of the Nile Valley and to the south of Egypt (Fig. 1). The area of northeastern Aswan is covered mainly by Phanerozoic rocks (sedimentary) set unconformably over basement rocks of Precambrian age^34,35^ (Fig. 2). The Precambrian units show up as heavily fractured gneissic granite hills and younger granite with a tonalitic to granodioritic makeup. The Cambrian-Upper Cretaceous Nubia sandstone (NSS) sequence is the sedimentary cover of the area, composed mainly of sandstones intercalated by siltstones and shale, as well as some iron oxides (e.g., hematite and goethite). The Nubia sandstone succession (Cambrian to upper Cretaceous) has been classified by Attia^36^ into three main parts: (i) the lower part is made of kaolin, and conglomeratic beds rest unconformably over the Precambrian rocks; (ii) the middle part is marked by oolitic iron ores and ferruginous beds; and (iii) the upper part is dominated by a thick quartzitic sandstone. Salem and El Gammal^17^ arranged the succession from the youngest to the oldest, as follows: Quaternary deposits and sand sheets were succeeded by the Quseir, Umm Brammily, Timsah, Abu Aggag, El Burg, Lake Nasser, and Abu Simbel formations. The study area includes the following lithologies: 1. The Precambrian assemblages (Fig. 2); 2. The Nubia succession, represented by the Abu Aggag, Timsah, and Umm Brammily formations (Fig. 3a).

The Abu Aggag and Umm Brammily formations cover the majority of the study area. The E-W-trending Wadi Abu Aggag traverses the Abu Aggag Formation, marking a sharp contact with the basement rocks (Fig. 3a). The central part of the study area, above the Abu Aggag Formation, exposes the Timsah Formation in small parts. According to Salem and El Gammal^17^, the Timsah Formation is identified as the primary origin of iron ore in the examined zone. The Timsah Formation consists of ferruginous sandstone beds. These beds are formed by a combination of fluvial, marine shore, and eolian processes. These beds also contain interbedded channels and soil deposits (Fig. 3b). Figure 2 records a red ferruginous faint that colors the outcrops of the three formations.

**Fig. 4:**
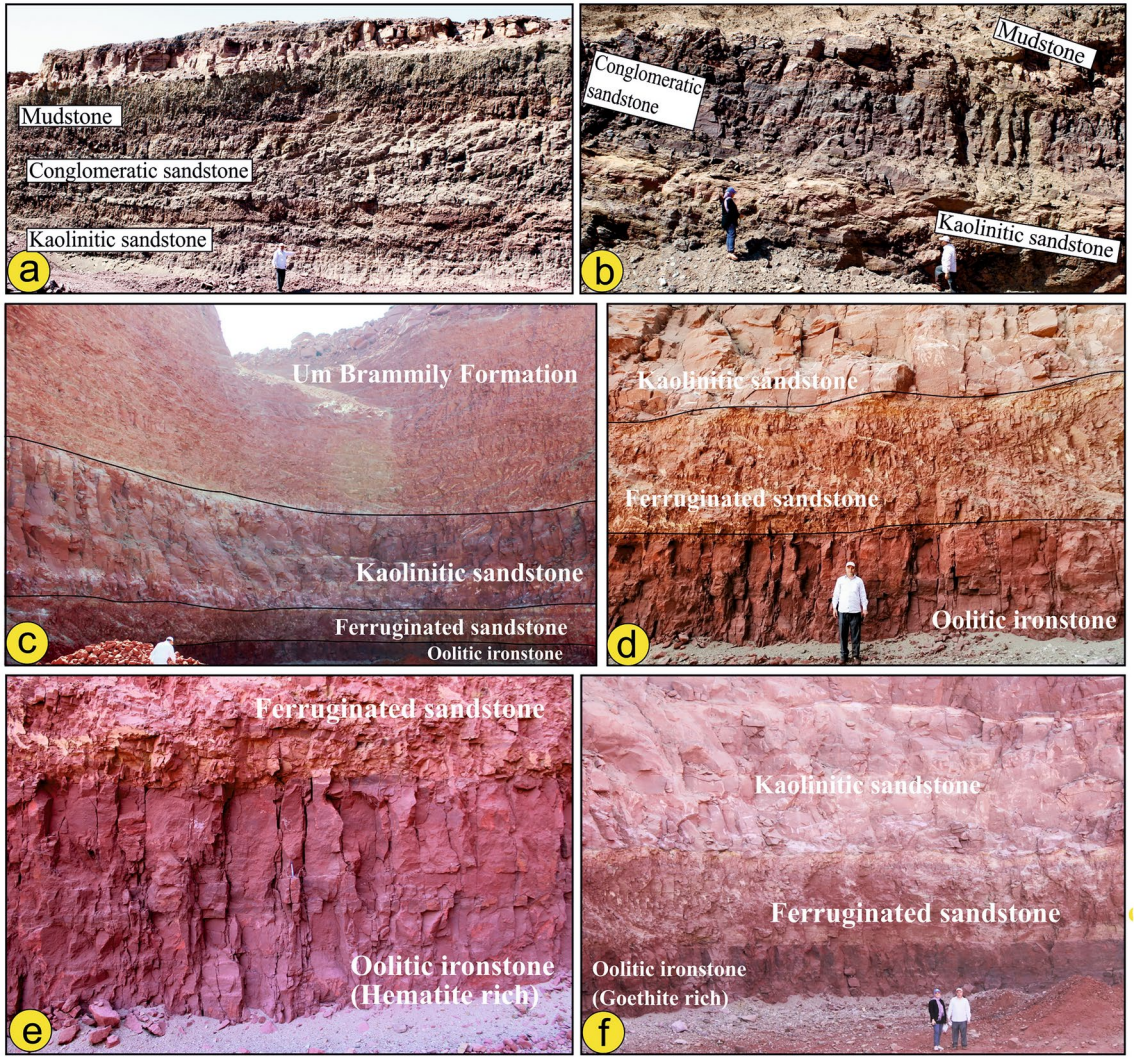
(**a**, **b**) showing Abu Aggag Formation composed of three successive units: (i) kaolinitic sandstone, (ii) conglomeratic sandstone and (iii) mudstone-dominated unit, (**c**, **d**) Timsah Formation in Wadi Abu Subeira is made up of rhythmically alternating and laterally intertonguing beds of kaolinitic and ferruginous sandstones, oolitic ironstone and mudstone overlain by sandstones and conglomerates of Umm Brammily Formation, (**e**, **f**) Oolitic sandy ironstone of Timsah Formation is found as brecciated bed vary in thicknesses from 2 to 2.5 m and enriched in dark red oolitic hematite (**e**) and goethite (**f**). The field photographs in figure are taken by the authors (Mohamed Abd El-Wahed and Dina Younes) of current research. These photos are our own and we agreed to publish them.

Our field investigations indicate that the Abu Aggag Formation is characterized by a significant fining-upward cycle that originated in a braided stream system. The Abu Aggag Formation is composed of three successive units: (i) At its base, the first unit is a kaolinitic conglomerate, primarily composed of channel lag deposits (Fig. 4a,b). This unit represents the accumulation of sediment in the distal channels of braided streams. (iii) Located at the top, the third unit primarily consists of mudstone, symbolizing the sedimentation of the floodplain (Fig. 4a,b). The Abu Agag Formation appears to be devoid of fossils, but it may contain some poorly preserved plant remains and root molds^37^.

Sandstone makes up the top portion of the Timsah Formation, while massive, laminated gray (light to dark, brownish to yellowish) and hard clays make up the lower portion. Beds of kaolinitic and ferruginous sandstones, oolitic ironstone, and mudstone, rhythmically alternating and laterally intertonguing (2–6 m), follow the basal mudstone-dominated unit. The sandstone is very difficult to indurate, exhibiting different structures (trough and cross-bedded) and sizes (e.g., fine to coarse-grained), with a wide range of colors.

Fluviatile sandstone makes up the Umm Brammily Formation. Fluvial clastics from the Um Brammily Formation were deposited in the area that was studied when the sea level dropped during the Santonian–Campanian period^38^. The Umm Brammily Formation is composed of a fining-upward sequence, beginning with channel lags at the base, followed by cross-bedded sandstones (Fig. 4c) and conglomerates. The sequence ends with fine-grained sandstones, siltstones, and a paleosol^38^. The ball clay in the Abu Subeira and Abu Aggag areas consists mainly of gray, beige, reddish-gray, and brownish-gray clays. Many ceramic and tile firms mine ball clay for both household and industrial usage.

### The Oolitic iron ore

The oolitic sandy ironstone of the Timsah Formation varies in thickness from 2 to 2.5 m and is enriched in dark red oolitic hematite and goethite (Fig. 4e,f). In some places, the main oolitic sandy ironstone is separated into two beds: the lower compact bed and the upper fine-grained strongly laminated bed. This ironstone bed ranges in composition from oolitic sandy ironstone to oolitic ironstone and consists of grain-supported quartz grains and less abundant ferruginous ooids. The oolites are bound together by pure amorphous hematitic material and ferruginous silica, resulting in a matrix with a lower iron concentration than the oolites. The oolitic hematitic grains are easily visible to the naked eye and come in varying sizes in different specimens and even within the same specimen. The true oolitic ironstone has been replaced by oolitic sandy ironstones or sheeted cross-bedded sandstone (Fig. 4e,f).

The oolitic sandy ironstone of the Timsah Formation is locally brecciated, where it is found as massive, sharp-angled shards contained in a fine-grained matrix of smaller particles. This brecciation is caused by faulting and fracturing. The Aswan iron ores are composed of various components such as cryptocrystalline hydrated hematite, microcrystalline hematite, clay minerals, quartz with subangular to subrounded form, goethite, and hydrogoethite. The matrix is more recrystallized than oolites because solutions flow through it more easily^39^. Anhedral detrital quartz grains may be observed distributed inside the hematitic matrix^40^. Hematite and goethite are secondary iron minerals that form through the weathering of primary iron-bearing minerals, such as magnetite and pyrite^40^.

## Remote sensing data and methods

### Remote sensing data

Cloud-free scenes of Landsat-8 and ASTER were acquired on March 10, 2023, and May 16, 2015, respectively. These datasets were obtained through the United States Geological Survey (USGS) and NASA Earth Data Center, to enhance the lithological mapping and explore the potential areas of iron ore deposits within the study area. The Landsat-8 satellite covers eleven bands with different spectral ranges and spatial attributes (Table 1) through two sensors, including the Operational Land Imager (OLI) and the Thermal Infrared Sensor (TIRS), while the ASTER includes 14 bands covering visible and near-infrared (VNIR), short-wave infrared (SWIR), and thermal infrared regions (TIRS), as shown in Table 1. According to the spectral characteristics of our target and the sensors utilized, VNIR and SWIR bands (B) of Landsat OLI (B2 to B7) and ASTER (B1 to B9) were employed to unravel the spatial distribution of iron oxide and identify the lithological units of the area under consideration. In addition to lithological discrimination, structural analysis was performed using radar data due to its superiority in extracting linear features over multispectral data^41^. The current research used the Sentinel-1B (S1B) scene, which was acquired on December 11, 2021 (S1B_IW_GRDH_1SDV_20211201T15 5425_20211201T155450_029834_038FB1_1B28), from https://search.asf.alaska.edu for automatic lineament extraction. The use of S1B data could improve the structural analysis by extracting the linear features in the study area and highlighting their influence on iron ore deposit allocation. The term S1B data refers to a synthetic aperture radar (SAR) instrument that operates in the C-band and can provide a spatial resolution ranging from less than 5 m to a swath width of over 400 km. The instrument can operate in either single polarization (VV or HH) for the wave mode or dual polarization (VV + VH or HH + HV). Table 2 summarizes the properties of each S1B mode.

**Table 1:** The characteristics of the used spectral bands of Landsat-8 OLI sensor and ASTER data (Irons et ail, 2012, Roy et al., 2014).

Landsat-8 OLI				ASTER			
Bands (B)	Wavelength (µm)	Spectral Region	Resolution (m)	Bands (B)	Wavelength (µm)	Spectral region	Resolution (m)
B2	0.452–0.512	B	30	B1	0.52–0.60	VNIR	15
B3	0.533–0.590	G		B2	0.63–0.69		
B4	0.636–0.673	R		B3	0.63–0.69		
B5	0.851–0.879	NIR		B4	1.60–1.70	SWIR	30
B6	1.566–1.651	SWIR		B5	2.145–2.185		
B7	2.107–2.294	SWIR		B6	2.185–2.225		
				B7	2.235–2.285		
–	–	–	–	B8	2.295–2.365		
–	–	–	–	B9	2.360–2.430		

*B* Blue, *G* Green, *R* Red, *NIR* Near Infrared, *SWIR* Short wave Infrared, *VNIR* Visible Nera Infrared.

**Table 2:** Highlighted the characteristics of Sentinel-1B modes. (Torres et al., 2017; Schubert et al., 2017).

Radar AM	Sentinel-1B			
	Stripmap (SM)	Interferometric wide swath (IW)	Extra wide swath (EW)	Wave (WV)
Beam mode	S1 to S6	IW1 to IW3	EW1to EW5	WV1&WV2
Center frequency	C-band (5.405 GHz)			
Polarization	SP (HH or VV) DP (HH + HV and VV + VH)			SP (HH or VV)
Spatial resolution (range × azimuth) (m)	5 × 5	5 × 20 m	25 × 100	5 × 20
Band width (Km)	80	250	4	20 × 20
Chirp bandwidth (MHz)	87.6–42.2	56.5–42.8	22.2–10.4	74.5 & 48.2
Incidence angle (deg)	20–43^◦^	30–42^◦^	20–44^◦^	23 & 36.5^◦^

*AM* Acquisition mode, *SP* Single Polarization, *DP* Daul Polarization, *deg* Degree.

### Methods

Besides the fieldwork and structural analysis, different techniques were applied to the remote sensing datasets to produce an enhanced geological map of the study area. Additionally, automatic extraction of lineaments and comprehensive structural analysis were performed and linked with our remote sensing indications to detect the favorable zones for iron ore deposits within the investigated area.

#### Pre-processing techniques of satellite datasets

The two scenes of ASTER data were mosaicked into a single scene covering the whole study area before applying further pre-processing techniques. All the scenes from Landsat 8, ASTER, and S1B were geometrically corrected and georeferenced to the UTM Zone 36 North projection using the WGS-84 datum. The atmospheric correction using the Internal Average Relative Reflection (IARR) technique was applied only to the Landsat-8 and ASTER scenes to reduce or remove the atmospheric effects; this could affect the quality of the results from the processing steps (e.g., image classification and mineral detection). Then, each of the six utilized bands of Landsat OLI data (B2 to B7) and the nine bands of ASTER data (B1 to B9) were stacked.

To reduce the speckle in the radar imagery of Sentinel-1B, the enhanced Lee filter was applied to both polarizations, VH, and VV while simultaneously conserving the texture evidence^41^. Moreover, the band math was performed to produce the VH + VV, which was then stacked with the two polarizations, VH, and VV, as a single layer. The satellite imagery of each of Landsat-8, ASTER, and Sentinel-1B is subsetted to show only the area under study.

#### Processing techniques of satellite datasets

Several image transformations, including FCCs, BRs, MNF, PCA, and decorrelation stretch, besides SAM as a spectrum matching technique, were applied to the satellite datasets for high-quality geological mapping and detection of iron oxide minerals in the study area. FCC is a helpful tool for lithological discrimination and tracking the wadis or lineaments (e.g., fractures, joints, and faults)^42,43^. Band rationing is an essential image enhancement tool that could be applied by dividing the pixels’ value of one band by the corresponding value for another band or by applying other mathematical operations^44^. BRs could be used for the lithological identification and exploration of ores/minerals-rich localities (e.g., iron ores, clay minerals, and sulfides), allowing the production of an index map for these minerals^17,45–47^.

In image processing, decorrelation stretch increases color variation within a color image, thereby improving and reducing redundancy in the images. Satellite data undergoes processing using PCA, a mathematical method, to generate uncorrelated output bands. The method effectively separates noise components and minimizes redundant information across different bands through a form of rotation. PCA is a feasible method used for creating multispectral images for geological interpretation and the detection of minerals. The current study primarily used PCA as a feature enhancement method to highlight lithological units and iron-rich zones, providing insights into the main structural elements. MNF, a common technique that involves applying two steps of PCA to multispectral imagery to reduce and separate noise in the data^48,49^, was considered a useful tool for lithological discrimination in this study.

The SAM method is a technique for calculating the spectral similarity of reference spectra collected from field or laboratory observations and spectra produced from satellite images. Kruse et al.^50^ originally proposed the use of this spectrum technique, and it has since been applied by researchers such as Abedi et al.^51^ and Abd El-Wahed et al.^49^. The SAM method relies on calculating the angles between the pixel spectra of the image and training data (ROIs) or library spectra of specific minerals.

This study used Landsat-8 and ASTER data as input and applied BR and PCA techniques as image transformations and SAM as a spectrum-matching technique to explore the iron ore minerals^52^ in the study area. Also, PCA was applied to S1B radar data to extract the lineaments and explore the area of high lineament density. The latter was then employed with the SAM results to produce a potential map of iron ore in the northeastern Aswan area. All the processing techniques were applied using a package of software such as ENVI (ENVI v. 5.6.2. software; https://www.l3harrisgeospatial.com/Software-Technology/ENVI) for the image classification and spectrum matching techniques, as well as PCI Geomatica 2016 and RockWork16 software for lineament extraction. The ArcGIS software (ArcGIS Desktop 10.8.: https://www.esri.com/en-us/arcgis/products/arcgis-desktop/overview) was employed for the all-satellite datasets.

## Results

### Lithological mapping based on Landsat-8 OLI

**Fig. 5:**
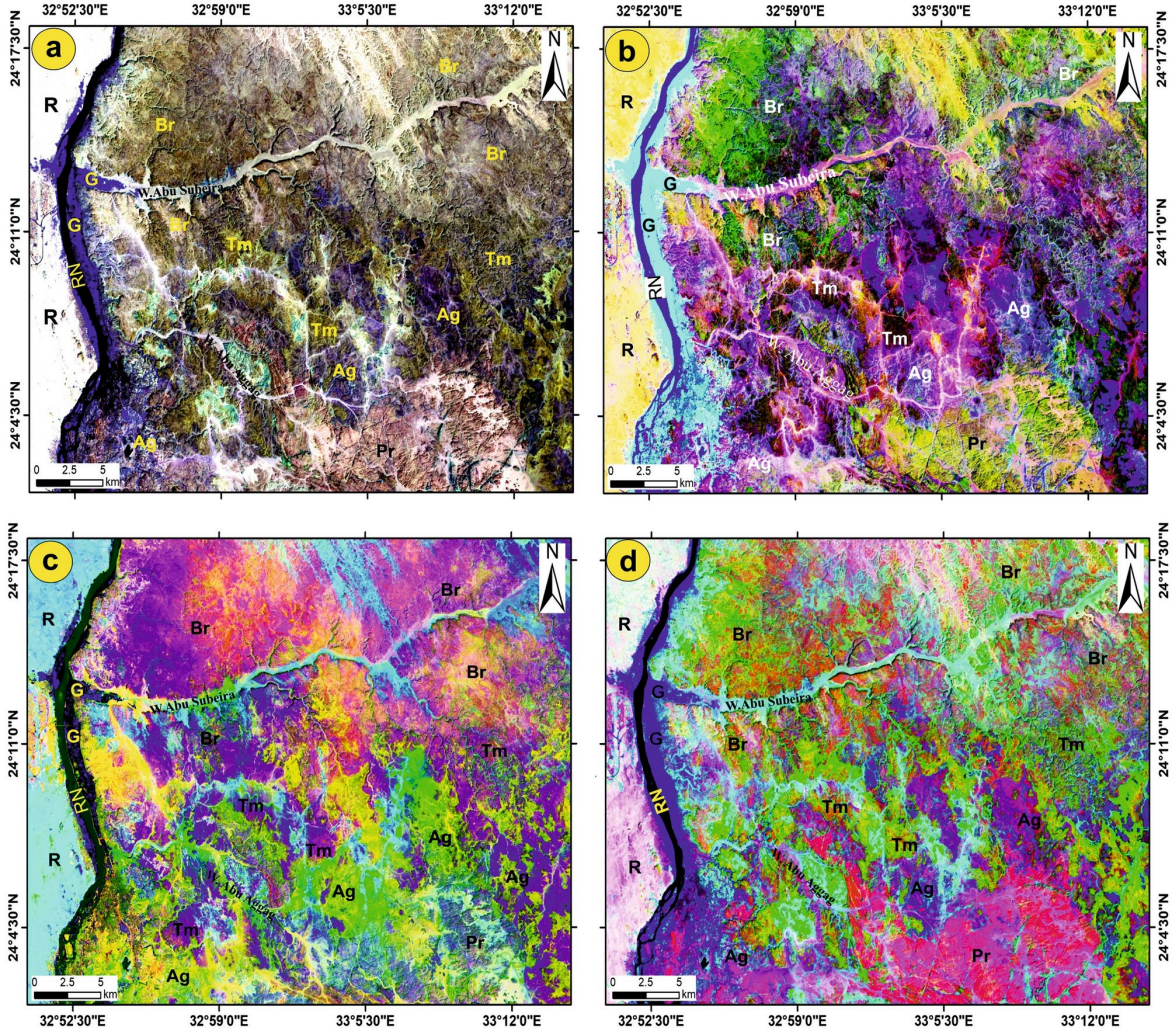
Demarcation of the lithological units of the study area using Landsat-8 OLI. (**a**) FCC 765-RGB; (**b**) BR 4/2, 5/4, 5/7-RGB; Decorrelated stretch of FFCs (**c**) 246 and (**d**) 765 in RGB. Recent deposits = R; Aggag Fm = Ag; Timsah Fm = Tm; Umm Brammily Fm = Br; Precambrian rocks = Pr; Green land area = G and River Nile = RN. (These images (Landsat 8 and ASTER) were downloaded through USGS. These figures were created and processed by ENVI v. 5.6.2. software: https://www.l3harrisgeospatial.com/Software-Technology/ENVI), which is mainly utilized for image processing, and 3-ArcGIS Desktop 10.8. (https://www.esri.com/en-us/arcgis/products/arcgis-desktop/overview/).

To provide additional information about lithological identification and to aid geological mapping in the study area, a variety of image classifications were applied to Landsat-8 data. The FCC 765-RGB gives us useful details for following the wadis, lineaments (like faults and joints), and lithological contacts between the different types of rock that are exposed (Fig. 5a). Salem and El Gammal^17^ applied the Landsat-8 BRs 5/7, 5/4, 4/2, and 4/2, 5/4, 5/7 in RGB to highlight the differences among the rock units and explore the iron ores east of Aswan. Therefore, they applied the BRs 4/2, 5/4, and 5/7 (Fig. 5b) to the studied area. The BRs 4/2, 5/4, and 5/7 (Fig. 5b) distinguish the Precambrian rocks with a greenish-yellow color, while the three formations exhibit different colors: Abu Aggag (Ag) Formation is in blue to light violet-blue, the Timsah Formation (Tm) is in reddish brown, and the Umm Brammily (Br) Formation is in green to deep violet. A decorrelation stretch was performed on FCCs 246-RGB and 765-RGB to enhance the different colors resulting from the original-colored composites. The decorated RGB images (246 and 765 in RGB) are successfully able to highlight and separate between the three formations and the basement rocks south of the considered area. The decorrelated image 246-RGB (Fig. 5c) identifies the Precambrian rocks as white blue, the Abu Aggag Formation as green to yellowish green, the Timsah Formation as pinkish deep violet, the Umm Brammily Formation as pinkish light violet, the green areas as deep blue, and the recent deposits as cyan. In the decorated image of 765 (Fig. 5d), the basement rocks display a pink hue, while the Abu Aggag Formation, Timsah Formation, and Umm Brammily Formation exhibit violet hues. The Abu Aggag Formation appears in a reddish-green color, whereas the Timsah and Umm Brammily formations are in a blue color, and the recent deposits are in a pinkish-bluish white color.

**Fig. 6:**
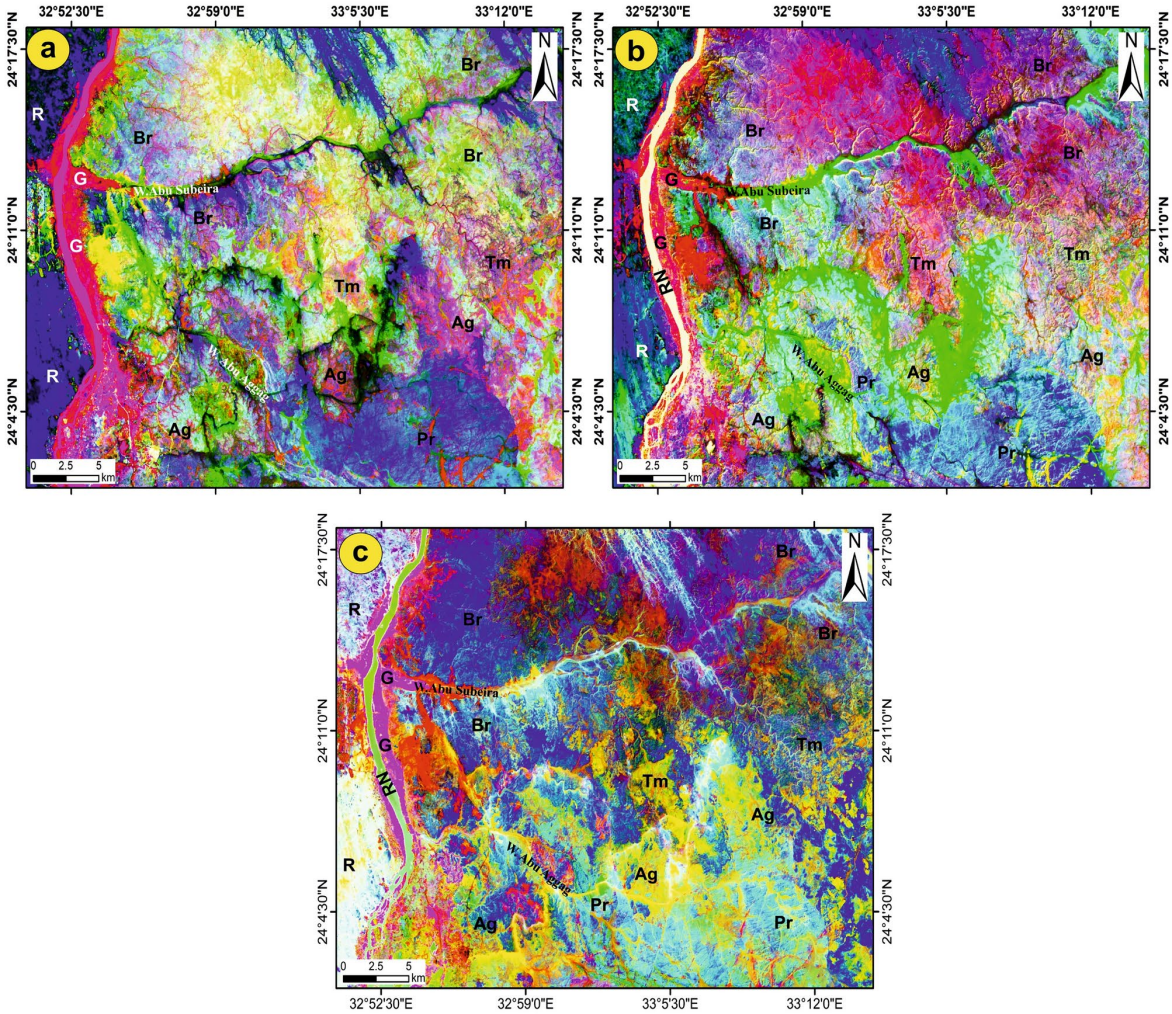
Lithological discrimination via MNF (**a**) 124 and (**b**) 134 in RGB; (**c**) PCA 243 in RGB of Landsat-8 OLI. (These images (Landsat 8 and ASTER) were downloaded through USGS. These figures were created and processed by ENVI v. 5.6.2. software: https://www.l3harrisgeospatial.com/Software-Technology/ENVI), which is mainly utilized for image processing, and 3-ArcGIS Desktop 10.8. (https://www.esri.com/en-us/arcgis/products/arcgis-desktop/overview/).

Moreover, the MNF and PCA transformations were carried out on Landsat-8 to help in the geological mapping. The MNF 124 and 134 in RGB (Fig. 6a,b) were found efficient for highlighting and enhancing the visualization of some rock units and the structural elements. MNF 124-RGB could successfully differentiate between the Precambrian assemblages in blue color, the Abu Aggag Formation, and Timsah Formation in purple to whitish purple color, and the Umm Brammily Formation appears in whitish blue color (Fig. 6a). The sharp contact between the highly jointed fractured basement rocks and Abu Aggag Formation was clearly visible, with the basement being marked by a shiny blue color, Abu Aggag Formation a by nearly white color, Umm Brammily Formation by a purple to bluish white color and Timsah Formation was exposed as a light purple to whitish purple color (Fig. 6b). In the PCA 123-RGB (Fig. 6c), Abu Aggag Formation appears in yellowish blue to shiny yellow pixels, Timsah Formation by a blue to deep yellow pixels, and Umm Brammily Formation appears in deep to light blue pixels, while the Precambrian units are marked by shiny cyan pixels.

### Iron ores distribution based on band rationing and SAM technique

#### Band Rationing

For iron mapping or demarcating iron ore mineral occurrences, several Landsat-8 and ASTER band ratios are applied for iron ore detection, like Landsat OLI 4/2^3,13,17,34,46^, 6/4 (ferrous iron oxides) and 4/3 (ferric iron oxides)^46,53^, 5/3 and 1/2 (for Fe^2+^)^21,46^. Ghoneim et al.^47^ proposed a new Landsat-8 band ratio of 6/2 based on the spectral signatures of iron minerals (e.g., hematite and limonite). This ratio was applied to highlight zones of higher iron content at the Gabal El-Hadid and Wadi Karim areas in the Central Eastern Desert of Egypt. The BR 4/2 of Landsat-8 was recommended as an effective ratio for iron exploration by Ghoneim et al.^47^.

**Fig. 7:**
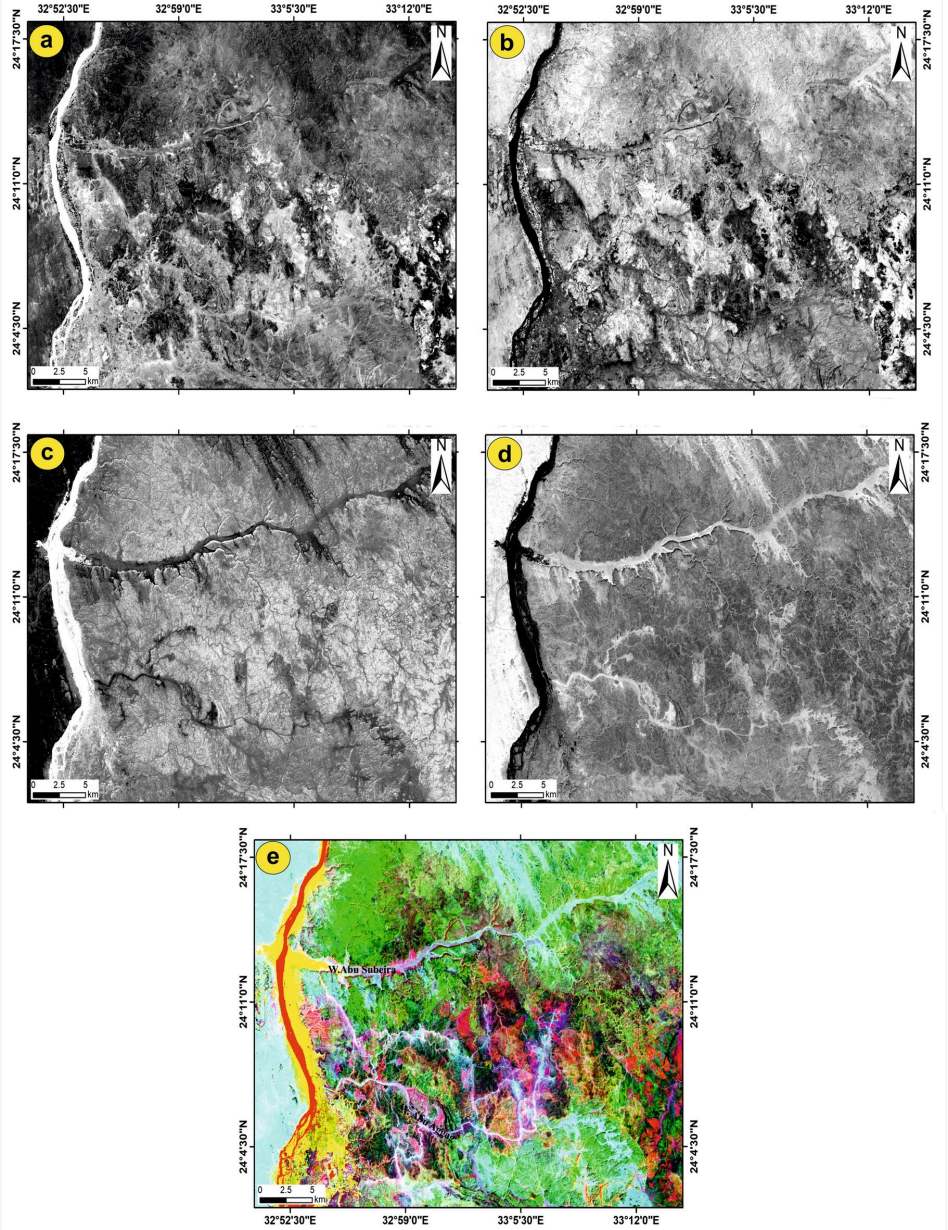
Allocation of iron oxides using band ratios. (**a**) BR 4/6 and (**b**) BR 6/4 in grey of Landsat-8 OLI highlighted the iron oxides as bright and dark pixels, respectively; ASTER BRs (**c**) 1/2 and (**d**) 2/1 in grey displayed the iron oxides as dark and bright pixels, respectively; (**e**) Landsat-8 BR 5/7 5/4 4/2 in RGB marked the iron minerals as pink patches. (These images (Landsat 8 and ASTER) were downloaded through USGS. These figures were created and processed by ENVI v. 5.6.2. software: https://www.l3harrisgeospatial.com/Software-Technology/ENVI), which is mainly utilized for image processing, and 3-ArcGIS Desktop 10.8. (https://www.esri.com/en-us/arcgis/products/arcgis-desktop/overview/).

The BRs (4/6), (6/4) in grey, and 5/7, 5/4, and 4/2 in RGB of Landsat OLI, as well as the ASTER BRs (1/2) and (2/1) in grey, were used as effective ratios to differentiate the iron oxides in the study area (Fig. 5). The Landsat-8 ratio 4/6 in grayscale (Fig. 7a) highlighted the iron oxides as bright pixels; in contrast, the ratio 6/4 in grayscale (Fig. 7b) demarcated the iron oxides as dark or black pixels, but it shows the same sites of iron oxides displayed by BR 4/6. The ratios of ASTER 1/2 and 2/1 (Fig. 7c,d) confirmed the same sites for iron oxides obtained from the Landsat-8 ratios, in which the iron oxides appear as dark pixels in ratio 1/2 (Fig. 5c) and as bright pixels in ratio 2/1 (Fig. 5d). The Landsat-8 ratio of 5/4, 4/2-RGB helped to separate the iron oxides, which showed up pink spots spread out in the study area (Fig. 7e), especially in the middle part south of W. Abu Subeira and north of W. Abu Aggag.

#### Spectral angle mapper (SAM)

**Fig. 8:**
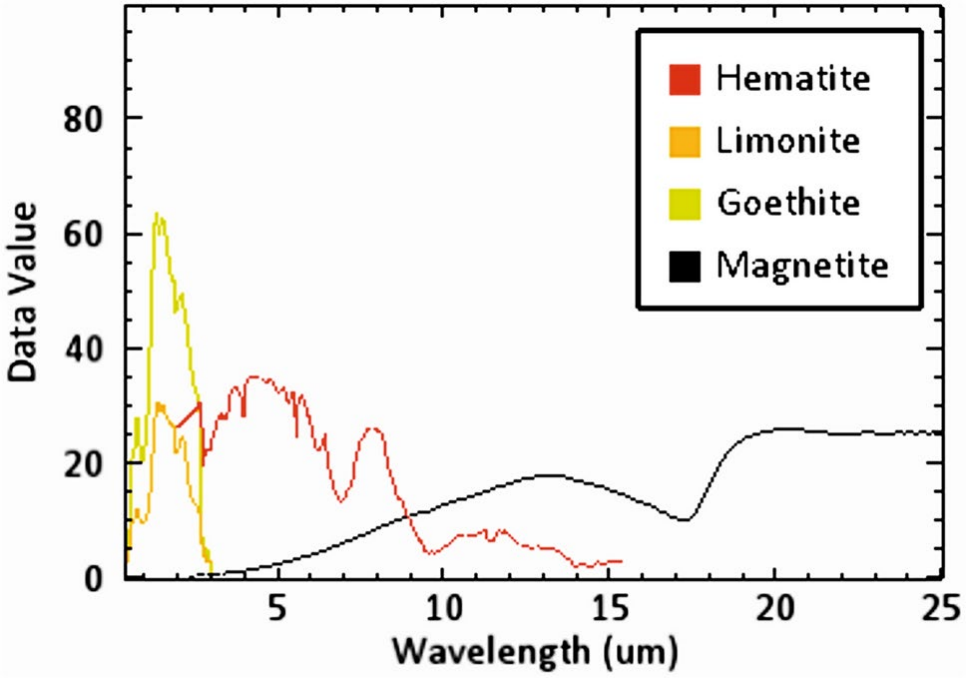
The spectral signature of the four iron oxide minerals sourced from USGS spectral libraries associated with the ENVI software (ENVI v. 5.6.2. software; https://www.l3harrisgeospatial.com/Software-Technology/ENVI).

**Fig. 9:**
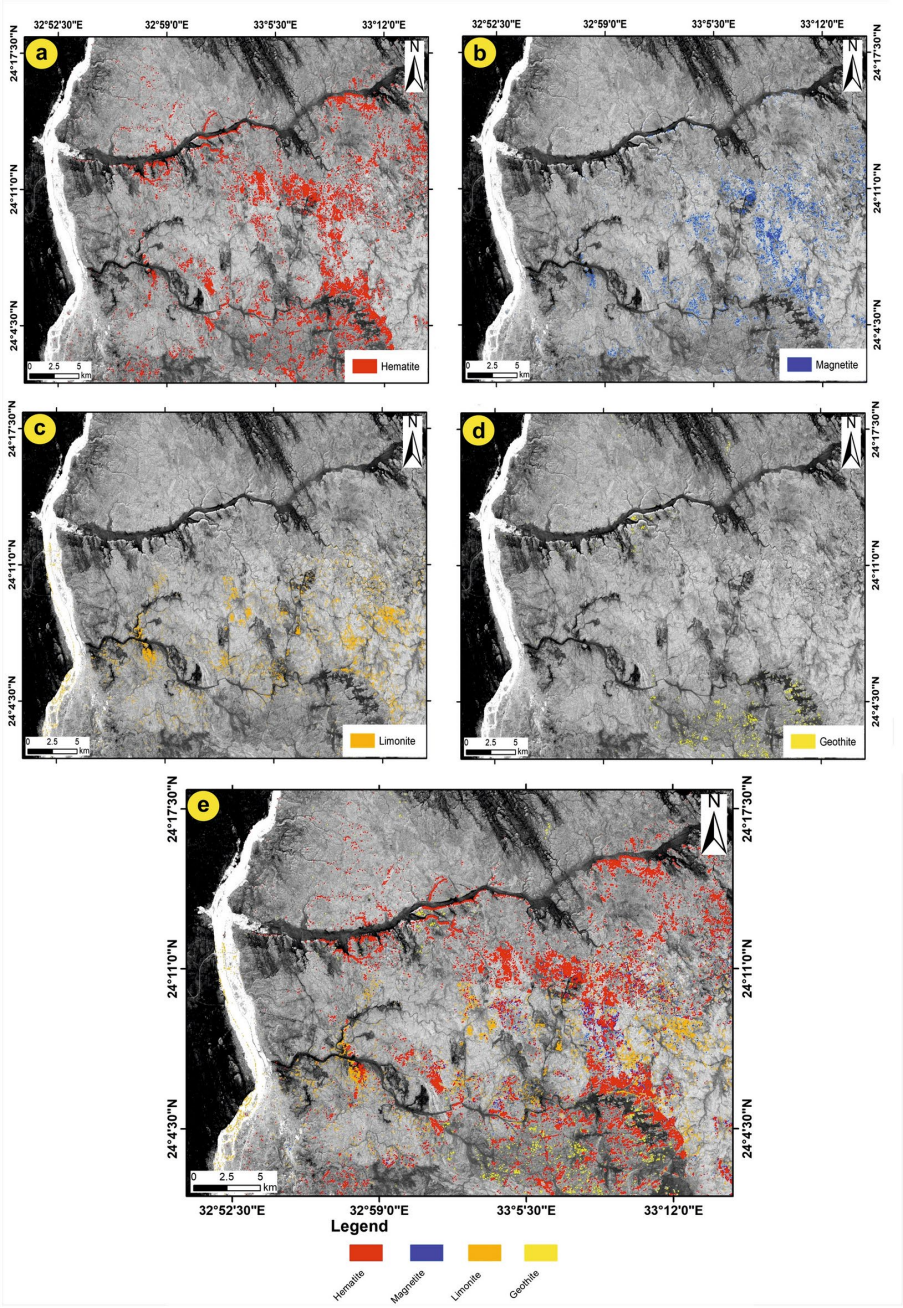
Allocation of the four iron oxide minerals using the SAM method. (**a**) Hematite; (**b**) Magnetite; (**c**) Limonite; (**d**) Geothite and (**e**) shows the distribution of the four iron oxides in the whole study area. (These images (Landsat 8 and ASTER) were downloaded through USGS. These figures were created and processed by ENVI v. 5.6.2. software: https://www.l3harrisgeospatial.com/Software-Technology/ENVI), which is mainly utilized for image processing, and 3-ArcGIS Desktop 10.8. (https://www.esri.com/en-us/arcgis/products/arcgis-desktop/overview/).

The algorithmic SAM method was applied to the nine bands of ASTER data to identify the distribution of iron oxide minerals, which are represented in the study area by hematite, magnetite, limonite, and goethite. To determine the similarity between a reference spectrum obtained from a spectral library and an unknown spectrum, the SAM technique computes the angle between two spectra by considering them as vectors in n-dimensional space. This estimation process is based on processing the spectra using mathematical techniques, as explained by Harris^54^. Using the Wizard detection of ENVI software (ENVI v. 5.6.2. software; https://www.l3harrisgeospatial.com/Software-Technology/ENVI) and based on the spectral signatures for the four iron oxide minerals (hematite, magnetite, limonite, and goethite; Fig. 8) sourced from the USGS spectral library, a series of processing steps were applied to the nine ASTER bands. The SAM software generates a collection of grayscale images, embellished with color depictions that highlight the most prominent regions of the iron minerals. To produce the SAM images of the four iron minerals, the rule threshold was moved to a certain value shown in Table 3, at which point each mineral started to appear, covering different areas with variable percentages (Fig. 9a–d).

**Table 3:** Statistic table show a brief summary of the SAM method for each mineral.

Mineral	Method	Rule threshold	Target count	Average area km^2^
Hematite	SAM	0.500	7949	2349.7766
Magnetite	SAM	0.320	3713	925.75409
Limonite	SAM	0.320	4704	928.89032
Geothite	SAM	0.320	726	879.54547

The distribution of iron minerals (Fig. 9a–d) closely aligns with the iron oxide sites highlighted by the Landsat-8 and ASTER BRs data. The SAM results for the four iron minerals, as well as the average area for each mineral (Table 3), show that hematite is the most common iron oxide in the study area. Limonite, magnetite, and goethite follow, each covering only small areas. All four iron minerals were displayed in a single image to manifest the distribution of iron oxides in the study area (Fig. 9e). The findings revealed a predominant distribution of iron oxides in the eastern part of the study area, extending from south to southeast of W. Abu Subeira, as well as in certain areas surrounding W. Abu Aggag.

#### Lineament extraction using Sentinel-1B (S1B) data

**Fig. 10:**
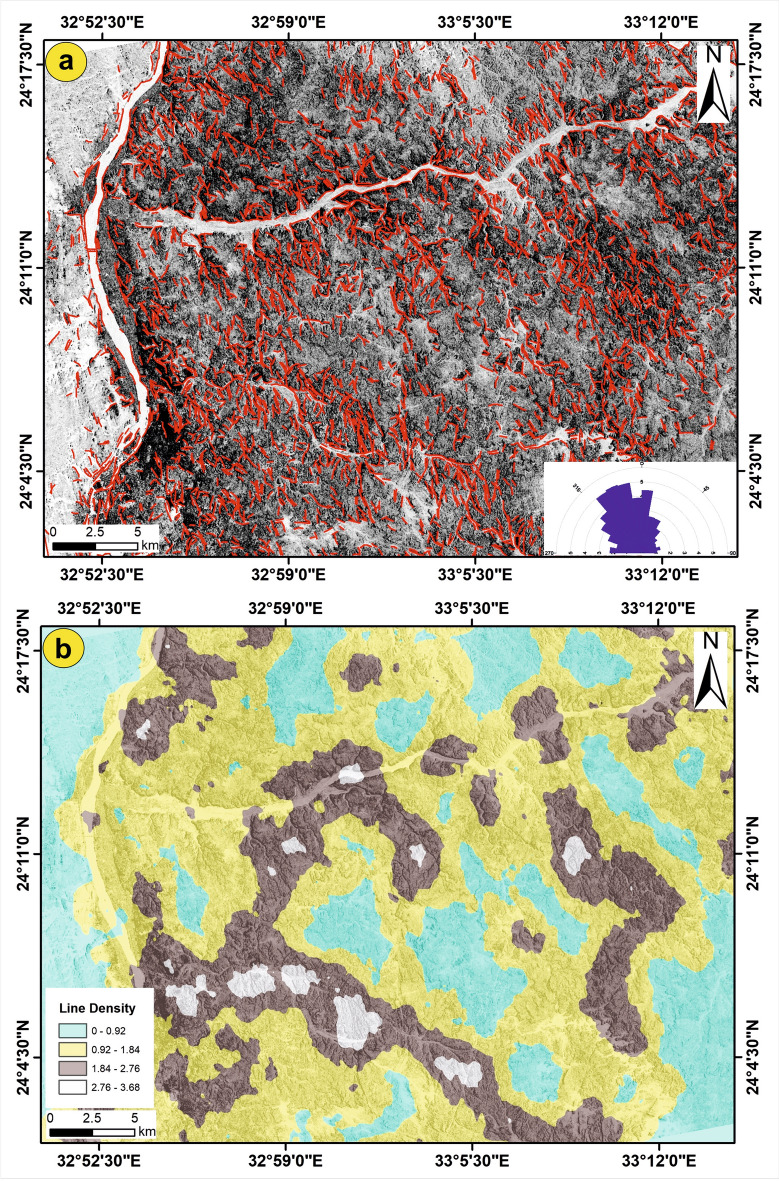
Automatically extracted lineaments using S1B data. (**a**) the extracted lineaments set over the PC1 in grey and (**b**) the line density map. (These images (Landsat 8 and ASTER) were downloaded through USGS. These figures were created and processed by ENVI v. 5.6.2. software: https://www.l3harrisgeospatial.com/Software-Technology/ENVI), which is mainly utilized for image processing, and 3-ArcGIS Desktop 10.8. (https://www.esri.com/en-us/arcgis/products/arcgis-desktop/overview/).

The PCA was carried out to transform the layer stacked (VH, VV, and VH + VV) S1B data to produce PC1, PC2, and PC3. As PC1 mostly involves the highest amount of information, PC1 was used and further processed for the automatic extraction of lineaments and constructing a lineament density map. The extracted lineaments (Fig. 10a) dropped over the gray image of PC1 were processed after that using RockWork16 to produce a frequency diagram highlighting the main trends within the study area (Fig. 10a). Excluding the areas covered by recent deposits, the Nile River, and green areas, the extracted lineaments were exposed as intensive red, short-length lines distributed and dissected nearly through all the rock units (Fig. 10a). According to the azimuth rose diagram (Fig. 10a), the most dominant trends of lineaments are NW–SE and NNW–SSE. Also, the NE–SW and E–W trends were recorded and observed in the rose diagram, but these two trends have a smaller domain than the NW trend. The density map (numbers per square kilometer; Fig. 10b) displays the density of the linear segments throughout the entire area. The density map shows that the lineaments are high to moderately concentrated along W. Abu Aggag, some parts along W. Abu Subeira, and the northern part of the basement units. The central and northern regions of the study area exhibit low lineament densities.

## Fault-related deformation

Besides the primarily automatic extraction of the linear features, further detailed structural analysis was performed to specify the deformational events affecting the study area and mostly controlled iron ore deposits through intensive field investigations and structural measurements. Our structural analysis revealed that the primary slip surfaces (fault zones) of the Abu Agagg and Umm Brammily formations have developed widespread fractures and wide rock cataclasis zones. The examined fracture network is composed of many fault sets. These fault sets can either be diverse, arising from the overprinting of two or more stress systems, or they can be conjugate, occurring within the same stress system^55,56^. This can lead to the formation of new faults with different orientations and the reactivation of existing faults^57,58^. Additionally, it can significantly influence the formation of subsequent fault sets^59^.

**Fig. 11:**
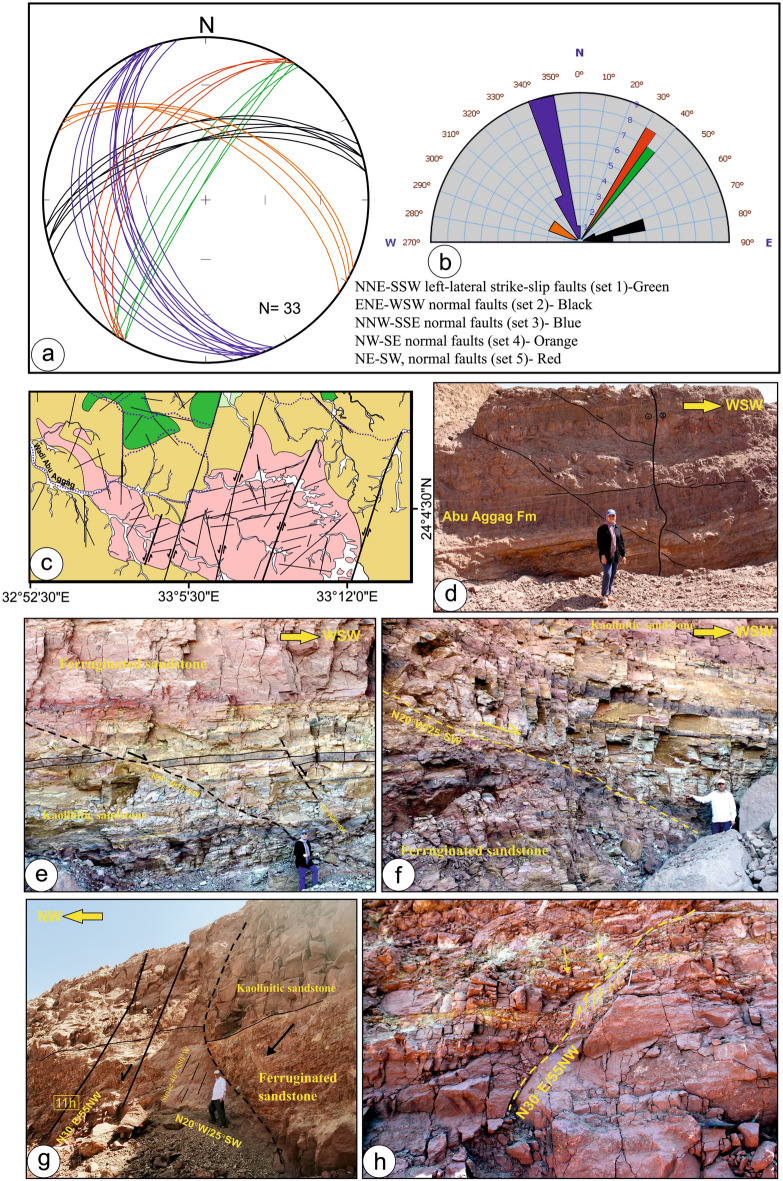
(**a**) Stereographic projections display the great circle of best-fit plane computed for each fault set, (**b**) Rose diagram showing orientation of the dominant fault set, (**c**) Geologic map showing en-echelon pattern of the NNE-SSW left-lateral strike-slip faults (set 1) in the Precambrian rocks and Abu Aggag Formation, (d) NNE-SSW left-lateral strike-slip fault in Abu Aggag Formation displaced by NNW-SSE striking normal faults (set 3), (**e**, **f**) NNW-SSE normal faults (set 3) in Timsah Formation from Wadi Abu Subeira, (**g**) Set 3 normal fault strike N20°W/45–55°SW with the slickenlines plunge moderately toward the SW (40°/S80°W) in Timsah Formation from Wadi Abu Subeira, (**h**) The fault hanging wall in (**g**) is displaced by another normal fault strike N30°E and dip 55° toward the NW (set 5). The field photographs in this figure are taken by the authors (Mohamed Abd El-Wahed and Dina Younes) of the current research. These photos are our own and we agreed to publish them.

Five primary fault sets were reported in the examined outcrops from Wadi Abu Subeira and Abu Aggag based on their orientation and kinematics (Fig. 2): sub-vertical, NNE-SSW left-lateral strike-slip faults (set 1); ENE-WSW normal faults (set 2); NNW-SSE normal faults (set 3); NW–SE normal faults (set 4); and NE-SW normal faults (set 5). Figure 11a and b display the stereoplots and rose diagrams of the main fault sets.

Set 1 sub-vertical strike-slip faults displace mostly Precambrian rocks and the Abu Aggag Formation and are characterized by apparent vertical offset (separations) ranging from a few millimeters to several meters. These faults had an en-echelon structure (Fig. 11c), and their left-lateral horizontal displacement (Fig. 11d) may be measured in kilometers (Fig. 2). Set 2 is made up of sub-vertical and ENE-WSW normal faults, mainly in the Precambrian rocks and Umm Brammily Formation. The main course of Wadi Abu Subeira is part of Set 2 normal faults. Set 2 faults are distinguished by displacements of a few meters. Set 3 is the most common normal fault in the study area. It is made up of NNW-SSE to N-S normal faults that move all the formations and have a big effect on the Timsah Formation (Figs. 11e–h). The course of the Nile River belongs to Set 3. Sets 2 and 3 are inner cataclastic fault cores that are made up of different types of rock fragments, cataclasites, and reddish clay (Fig. 11f). Both set 2 and set 3 fault cores have pockets of breccias consisting of large subangular sandstone rock clasts. These clasts are bound together by reddish, hematite-bearing laminae.

One of the set 3 normal faults strikes N20°W/45–55°SW (Fig. 11g) with the slickenlines plunging moderately toward the SW (40°/S80°W). This fault exhibits two unique features: a fan-shaped bed-parallel stylolite near the main fault plane and thicker beds in the fault hanging walls.. Both characteristics are clear signs of syn-sedimentary fault activity. The vertical deviations along these faults range from a few tens of centimeters to one meter. Also, the fault hanging wall of this Set 3 normal fault is displaced by another normal fault (Fig. 11h) that strikes N30°E and dips 55° toward the NW (Set 5). Set 4 faults are present mainly within the Umm Brammily Formation and dip from 50° to 70° to either SW or NE; their offset is up to a few meters. Set 4 faults crosscut Set 3 and, markedly, both Set 4 and Set 3 are crosscut by Set 5 normal faults that strike mainly NE-SW and dip toward the NW (Fig. 11g,h). Also, the NE-SW faults crosscut the NNE-SSW left-lateral strike-slip faults.

The displacements associated with the set 3 normal faults significantly impacted the ironstone beds, with the majority pointing westward. This increased the sedimentary load over the iron ore-bearing beds, as well as the cost of extraction. Furthermore, the movement of the faults to the west resulted in the absence of iron exploitation areas at the entrances to Wadi Abu Subeira and Abu Aggag, which would have increased the depth of the ironstone beds.

Fractures associated with the strike-slip faults (set 1) have an NNE–SSW and ENE–WSW orientation, running parallel and at low angles to the faults. On the other hand, fractures associated with normal faults strike in the E–W, NW–SE, and NE–SW directions.

## Discussion

### Potentiality of iron ore occurrences

**Fig. 12:**
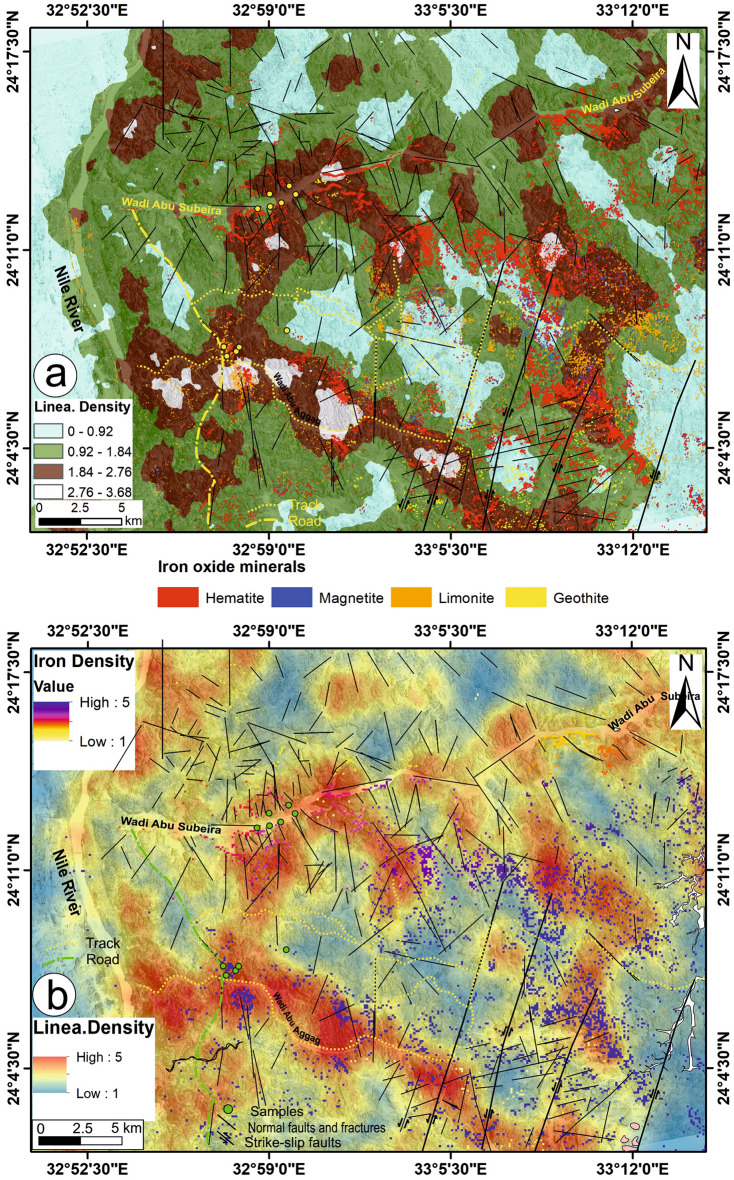
(**a**) Distribution of the four iron oxide minerals set over a lineament density map, (**b**) A potential map shows the area of high potentiality for iron exploration using a scale starting from 1 (as a low potentiality) and ending with 5 (as a high potentiality). (These images (Landsat 8 and ASTER) were downloaded through USGS. These figures were created and processed by ENVI v. 5.6.2. software: https://www.l3harrisgeospatial.com/Software-Technology/ENVI), which is mainly utilized for image processing, and 3-ArcGIS Desktop 10.8. (https://www.esri.com/en-us/arcgis/products/arcgis-desktop/overview/).

Figure 12a demonstrates a correlation between the presence of iron oxides and the occurrence of lineaments with moderate to high densities. Consequently, the lineaments have a significant influence on the location and distribution of iron ore deposits within the studied region. The assertion is consistent with the research conducted by Mekkawi et al.^12^, which determined that the presence of large faults and the movement of hydrothermal solutions along these faults in the shallow region impacted the location of iron ore deposits in W. Abu Subeira. As a result, we developed a prospective map to investigate the potential locations of iron ores. This was done by merging the distribution of the four iron oxide minerals with the density map of lineaments. Faults and fractures have a significant influence on the distribution of iron ores and serve as favorable pathways for iron-rich fluids, which are believed to be a primary source of iron in the studied region.

Using a suitable scale or range, start from 1 to mark the area with low iron ores and end at 5 to mark the areas with high content of iron ores (Fig. 12b); another range to characterize the density of linear segments also starts from 1 as low density and ends at 5 as high density of linear segments. So, according to the proposed iron potential map for the study area, it can be said that the areas shaded by blue colors (maximum range = 5) have a high potentiality for iron ore occurrences that are located southeast of W. Abu Subeira and along W. Abu Aggag, particularly in the Timsah and Aggag formations, where the density of linear segments is high (maximum range = 5). The areas shaded by yellow (minimum range = 1), where the density is low, are characterized by low iron ore content and are located mainly north of W. Abu Subeira in the Umm Brammily Formation, as well as in little sections in the southern part of the considered area (Fig. 12b).

### Tectonic significance of fault trends

**Fig. 13:**
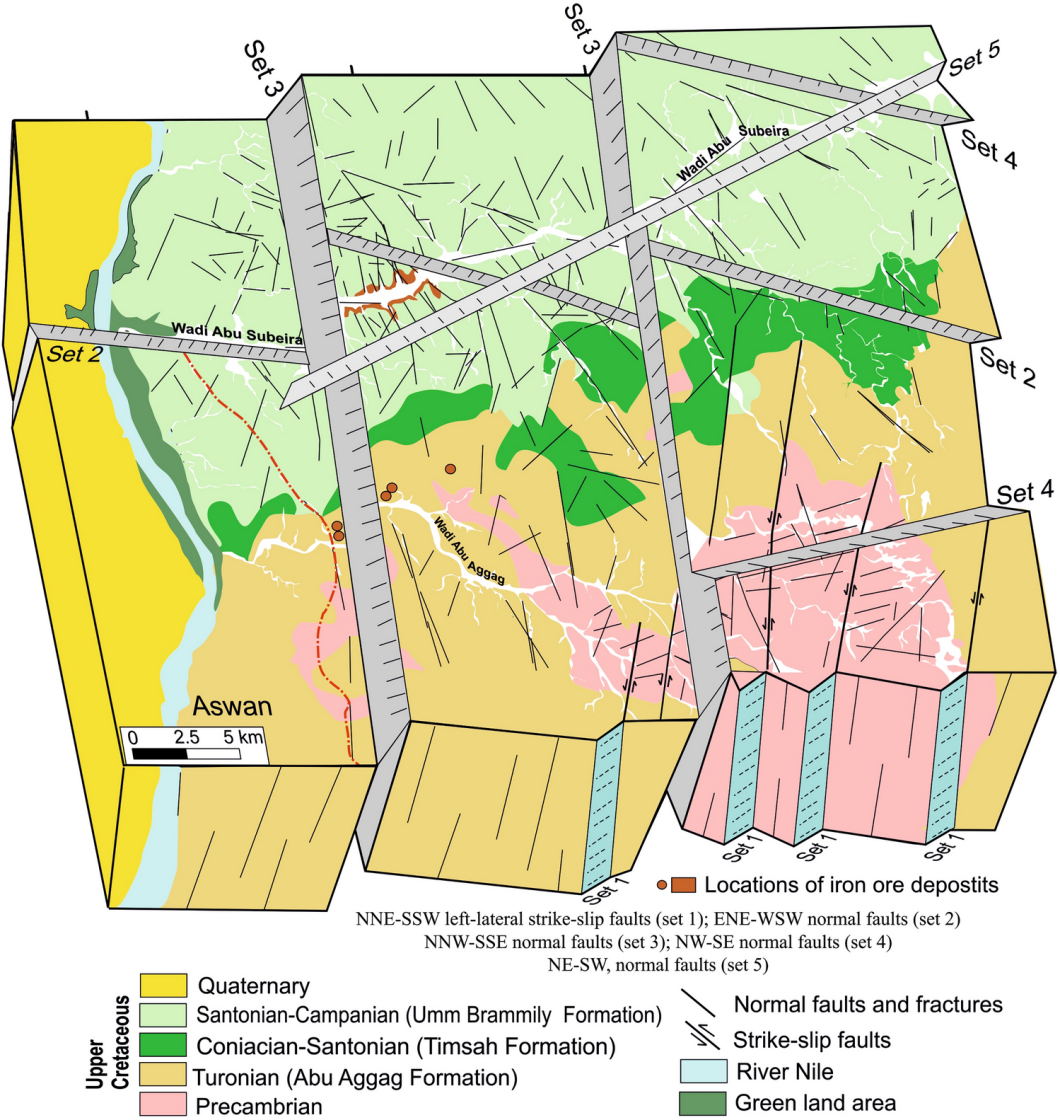
Block diagram shows the relation between the different fault sets in the Abu Subeira-Abu Aggag area.

The documented five sets of faults can be categorized into groups based on their characteristics (Fig. 13) that are kinematically compatible with major structure trends described by Meshref^60^ and Khedr et al.^61^. The sub-vertical, NNE-SSW left-lateral strike-slip faults (set 1) are created syn-genetically with the Gulf of Aqaba (N15°5°E) and Gulf of Aden (N70°5°E) trends. The sub-verticals, ENE-WSW normal faults (set 2), are probably related to the Guinean-Nubian lineament (80° ± 5°) NE trend^61^. The genesis of the Red Sea is connected to the emplacement of peralkaline rocks in Egypt due to their alignment along two fault lines: the Guinean-Nubian lineament that runs in an ENE-WSW direction and the Trans-African shear zone that runs in an NNW-SSE direction^62^. The Guinean-Nubian lineament, affecting the southern part of Egypt, ages from the Triassic to the recent past^63^. This is evidenced by the occurrence of rhyolites filling fault planes running NE 80° ± 5° as well as several orogenic alkaline complexes in the southern part of the Abu Subeira area ^64^. The reactivation of ancient ENE-trending shear zones in the Precambrian rocks^65–67^ is what causes the ENE-WSW trend in the Mesozoic rocks from the Abu Subeira-Abu Aggag area (Fig. 13).

The primary fault types in the area under investigation (Fig. 13) are the NNW-SSE normal faults, which are associated with the N20° ± 5°W trend of the Red Sea. This trend is believed to have occurred during the initial stage of the Red Sea rifting^61,68–71^. The N20° ± 5°W trend (Fig. 13) most likely developed because of tensional forces that likely began in the late Precambrian^72^, persisted during the Carboniferous because of depression, and lasted until the Cenomanian to the early Tertiary^61,73,74^. The ancient Red Sea rift in the Gulf of Suez formed during the Late Oligocene–Miocene period^75,76^. The left-lateral Dead Sea Transform, which separated the two rifts from each other during the Late Miocene, allowed the Red Sea basin to continue opening. Highly extensional normal faults (Fig. 13) characterize the Abu Subeira-Abu Aggag area, striking NNW–SSE (set 3) and NW–SE (set 4), parallel to the Gulf of Suez trend (N35°–45°W). The dominant tectonic setting in the Gulf of Suez is dominated by the major fault system, the NW–SE longitudinal faults (Clysmic trend)^77^. These sets align with the tectonism of the Suez Gulf-Red Sea and the Clysmic faults of the mid-tertiary.. The Trans-Red Sea Trend (N40°–50°E) aligns with the NE-SW normal faults (set 5).

### The ferruginous concretion of iron ore

Two main theories have been previously postulated for the genesis of oolitic ironstones^37^: (i) sedimentary origin^11,36,78^ and (ii) volcanogenic origin^79^. The iron ooids of Aswan ironstones are suggested to be formed in sedimentary basins from land-derived iron-rich constituents. However, Schwarz and Germann^80^ suggested that the iron ooids were formed during the lateritization of the hinterlands and then transported into the marine basin, where deposition took place. The volcanic origin for the oolitic ironstones is postulated by Tosson^79^, who proposed the derivation of iron from nearby volcanoes and its deposition in shallow marine environments. Other studies on the genesis, mechanism of ooid formation, paleoenvironments, and diagenesis of Aswan ironstones can be found in numerous publications^11,37,40,81^. The oolitic iron ores from Aswan are of marine (hydrogenous) origin, generated in open space along the sediment–water interface by the accretion of FeO, SiO_2_, Al_2_O_3_, and tiny quantities of other oxides around solid particles such as quartz and fractured ooliths^82^. The iron ores in Aswan, represented by hematite, magnetite, limonite, and goethite, occur as ironstones. Salem and El Gammal^17^ classified the iron ores in Aswan into (i) ferruginous sandstone iron ores, (ii) oolitic iron ore, and (iii) ferruginous concretion iron ore. They also reported that the Fe_2_O_3_ content in the ferruginous sandstones is estimated to be about 70.46%, while in the oolitic iron ore, the Fe_2_O_3_ content reaches 54%. Salem and El Gammal^17^ returned the occurrence and distribution of the three iron ore types to lithostratigraphy and morphotectonics.

Even though oolitic iron ore has a low content of Fe_2_O_3_ because of silica cement relative to the other types, it is considered the most dominant and valuable iron ore type in the study area. It occupies the upper part of the Timsah Formation, which lies south of W. Abu Subeira and occurs as dark red, compact oolitic hematite beds with a thickness of up to 3 m. While the iron ore of the ferruginous sandstone type occupies the lower parts of the Timsah Formation south of W. Abu Subeira, it occurs as inliers and caps of hematite and goethite strata, including limonite patches, within the Nubia sandstone beds with thickness varying from 50 cm to 4 m. This type of iron ore is marked by some common gangue minerals (e.g., quartz, gypsum, glauconite, and clay minerals) and is considered a syn-genetic bedded of Senomanian deposits that developed under a lacustrine environment^17^. Youssef^83^ reported that the ore deposits are found in two beds (B1) and (B2) in W. Abu Subeira and its surroundings, separated by ferruginous sandy clay and located at a depth of approximately 80 m. The ferruginous concretion iron ore is an accumulation of ferruginous sandstone, oolitic iron ore, and ferruginous concretion. It is exposed as thin ferric surfaces (hematite and goethite) of thicknesses ranging from 10 to 60 cm between the Nubia sandstone, forming hard, compact masses of concretion beds and substratum rich in iron ore along W. Abu Aggag.

## Conclusions

This study integrated multi-source remote sensing data, field investigations, and detailed structural analysis for a preliminary exploration of the iron-ore potential zones of NE Aswan, Eastern Desert, Egypt. The study concluded the following:

- The sedimentary cover of the area is the Cambrian-Upper Cretaceous NSS sequence, composed mainly of sandstones intercalated by siltstones and shale, as well as some ironstones. Abu Aggag, Timsah, and Umm Brammily formations cover the surface of the research region. The Abu Aggag Formation is made up of huge kaolinitic conglomerate, conglomeratic sandstone, and mudstone. The upper section of the Timsah Formation is composed of ferruginous sandstones, oolitic ironstone, and mudstone. Fluviatile sandstone makes up the Umm Brammily Formation.
- The Aswan iron ores consist of hydrated hematite with a cryptocrystalline structure, microcrystalline hematite, clay minerals, quartz, and goethite. The oolitic sandy ironstone of the Timsah Formation is found as a brecciated bed with a thickness of 2–2.5 m and is rich in dark red oolitic hematite and goethite. This ironstone is composed of grain-supported quartz grains and less numerous ferruginous ooids and its composition ranges from oolitic sandy ironstone to oolitic ironstone.
- The Landsat-8 ratio of 5/4, 4/2-RGB was useful in distinguishing the iron oxides as pink patches spread across the research region, particularly in the central sector south of W. Abu Subeira and north of W. Abu Aggag. The distribution of iron minerals is largely comparable with the same iron oxide locations indicated by Landsat-8 and ASTER BRs. Most of the iron oxides are located in the eastern part of the research area, from south to southeast near Abu Subeira and in certain locations near Abu Aggag.
- The two most prevalent lineament trends are NW–SE and NNW–SSE. The NE–SW and E–W trends were also documented and highlighted in the rose diagram, although their domains are less than the NW trend.
- The current research strongly recommends integrating various remotely sensed data, e.g., PCA, Band ratios, and SAM images, with the lineaments derived from radar data to map the distribution of the iron oxides within the study area (mainly correlated with the highly dissected zones).
- Five main fault sets govern the growth of the ironstone beds, including left-lateral strike-slip faults oriented in a NNE-SSW direction (set 1), normal faults oriented in an ENE-WSW direction (set 2), normal faults oriented in a NNW-SSE direction (set 3), normal faults oriented in a NW–SE direction (set 4), and normal faults oriented in a NE–SW direction (set 5). The intersection between Sets 2, 3, and 4 faults primarily controls the exposed ironstone beds along Wadi Abu Subeira, while Set 3 faults control those at Wadi Abu Aggag. The westward displacements of the Set 3 normal fault increased the depth of the ironstone beds and the thickness of the sedimentary overburden. This illustrates the lack of iron exploitation zones around the entrances of Wadi Abu Subeira and Abu Aggag, as well as to the west of the Nile River.

## Data Availability

The datasets used and/or analyzed during the current study are available from the corresponding author upon reasonable request.

## References

[1] Ranjbar, H., Honarmand, M. & Moezifar, Z. Application of the Crosta technique for porphyry copper alteration mapping, using ETM+ data in the southern part of the Iranian volcanic sedimentary belt. *J. Asian Earth Sci.* **24**(2), 237–243. 10.1016/j.jseaes.2003.11.001 (2004).

[2] Chen, X., Mao, J. & Tian, H. J. S. Analysis of China’s iron trade flow: Quantity, value, and regional pattern. *Sustainability* **12**(24), 10427. 10.3390/su122410427 (2020).

[3] Soydan, H., Koz, A. & Düzgün, H. Ş. Secondary iron mineral detection via hyperspectral unmixing analysis with sentinel-2 imagery. *Int. J. Appl. Earth Observ. Geoinform.* **101**, 102343. 10.1016/j.jag.2021.102343 (2021).

[4] Mohamed, M. T., Al-Naimi, L. S., Mgbeojedo, T. I. & Agoha, C. C. Geological mapping and mineral prospectivity using remote sensing and GIS in parts of Hamissana, Northeast Sudan. *J. Pet. Explor. Prod. Technol.* **11**(3), 1123–1138. 10.1007/s13202-021-01115-3 (2021).

[5] Egyptian Mineral Resource Authority (EMRA), Concession, Project # 1738, internal report, Abbassia, Cairo-Egypt (2015).

[6] Botros, N. S. & Noor, A. M. Mineral deposits in the Eastern Desert of Egypt, an expression of two major episodes with distinct magmatic and tectonic characteristics. *Ann. Geol. Surv. Egypt* **30**, 249–274 (2008).

[7] Abouzeid, A. M. & Khalid, A. M. Mineral industry in Egypt-part I: Metallic mineral commodities. *Nat. Resourc.* **2**, 35–53. 10.4236/nr.2011.21006 (2011).

[8] Hussein, A. A. Mineral deposits of Egypt. In *Geology of Egypt* (ed. Said, R.) (Bakema Publ Co, 1990).

[9] Mucke, A. Environmental conditions in the Late Cretaceous African Tethys: conclusions from a microscopic-microchemical study of ooidal ironstones from Egypt, Sudan and Nigeria. *J. Afr. Earth Sci.* **30**(1), 25–46 (2000).

[10] Salem, S. M., El Gammal, E. A. & Soliman, N. M. Morphostructural record of iron deposits in paleosols, cretaceous Nubia Sandstone of Lake Naser basin, Egypt, Western Desert, Egypt. *Egypt. J. Remote Sens. Space Sci.* **16**, 71–82 (2013).

[11] Bhattacharyya, D. Concentrated and lean oolites: Examples from the Nubia Formation at Aswan, Egypt, and significance of the oolite types in ironstone genesis. *Geol. Soc. Lond. Spec. Publ.* **46**, 93–103 (1989).

[12] Mekkawi, M. M. et al. Integrated geophysical approach in exploration of iron ore deposits in the North-eastern Aswan-Egypt: A case study. *Arab. J. Geosci.* **14**, 721. 10.1007/s12517-021-06964-0 (2021).

[13] Shebl, A. et al. Towards better delineation of hydrothermal alterations via multi-sensor remote sensing and airborne geophysical data. *Sci. Rep.* **13**(1), 7406 (2023). 37149689 10.1038/s41598-023-34531-y PMC10164183

[14] Shebl, A. & Hamdy, M. Multiscale (microscopic to remote sensing) preliminary exploration of auriferous-uraniferous marbles: A case study from the Egyptian Nubian Shield. *Sci. Rep.* **13**(1), 9173 (2023). 37280294 10.1038/s41598-023-36388-7 PMC10244429

[15] Shebl, A. et al. Novel comprehensions of lithological and structural features gleaned via sentinel 2 texture analysis. *Ore Geol. Rev.* **168**, 106068. 10.1016/j.oregeorev.2024.106068 (2024).

[16] Tangestani, M. H. & Moore, F. Comparison of three principal component analysis techniques to porphyry copper alteration mapping: A case study, Meiduk area, Kerman, Iran. *Can. J. Remote Sens.* **27**, 176–182 (2001).

[17] Salem, S. M. & El Gammal, E. A. Iron ore prospection East Aswan, Egypt, using remote sensing techniques. *Egypt. J. Remote Sens. Space Sci.* **18**, 195–206. 10.1016/j.ejrs.2015.04.003 (2015).

[18] Azizi, M., Saibi, H. & Cooper, G. R. J. Mineral and structural mapping of the Aynak-Logar Valley (Eastern Afghanistan) from hyperspectral remote sensing data and aeromagnetic data. *Arab. J. Geosci.* **8**(12), 10911–10918. 10.1007/s12517-015-1993-2 (2015).

[19] Mogren, S., Saibi, H., Mukhopadhyay, M., Gottsmann, J. & Ibrahim, E. Analyze the spatial distribution of lava flows in Al-Ays Volcanic Area, Saudi Arabia, using remote sensing. *Arab. J. Geosci.* **10**(6), 133. 10.1007/s12517-017-2889-0 (2017).

[20] Saibi, H. et al. Applications of remote sensing in geosciences. In *Recent Advances and Applications in Remote Sensing Book* (eds Hung, M.-C. & Wu, Y.-H.) 181–203 (Intech, 2018).

[21] Gopinathan, P. et al. Mapping of ferric (Fe3+) and ferrous (Fe2+) iron oxides distribution using band ratio techniques with ASTER data and geochemistry of Kanjamalai and Godumalai, Tamil Nadu, South India. *Remote Sens. Appl. Soc. Environ.* **18**, 100306. 10.1016/j.rsase.2020.100306 (2020).

[22] Guha, A. et al. Eigen vector based analysis of Landsat OLI principal components and constrained energy minimization maps for discriminating iron enriched zones in banded iron formation (BIF) in Sidhi, Madhya Pradesh. *Geocarto Int.* **37**(7), 1880–1898. 10.1080/10106049.2020.180186 (2020).

[23] Gad, S. & Kusky, T. ASTER spectral ratioing for lithological mapping in the Arabian-Nubian Shield, the Neoproterozoic Wadi Kid area, Sinai, Egypt. *Gondwana Res.* **11**, 326–335. 10.1016/j.gr.2006.02.010 (2007).

[24] Omer, E. A. H. & Zeinelabdein, K. A. Digital image processing of Landsat 8 and spectral analysis of ASTER data for mapping alteration minerals Southern Hamisana NE Sudan. *Al Neelain J. Geosci.* **2**(1), 10–20 (2018).

[25] Kalinowski, A. & Oliver, S. ASTER mineral index processing manual. *Remote Sens. Appl. Geosci. Austral.* **37**, 1–36 (2004).

[26] Azizi, H., Tarverdi, M. A. & Akbarpour, A. Extraction of hydrothermal alterations from ASTER SWIR data from east Zanjan, northern Iran. *Adv. Space Res.* **46**(1), 99–109. 10.1016/j.asr.2010.03.014 (2010).

[27] Hosseinjani, M. & Tangestani, M. H. Mapping alteration minerals using sub-pixel unmixing of ASTER data in the Sarduiyeh area, SE Kerman, Iran. *Int. J. Dig. Earth* **4**(6), 487–504 (2011).

[28] Pal, S. K., Majumdar, T. J., Bhattacharya, A. K. & Bhattacharyya, R. Utilization of Landsat ETM+ data for mineral-occurrences mapping over Dalma and Dhanjori, Jharkhand, India: An advanced spectral analysis approach. *Int. J. Remote Sens.* **32**(14), 4023–4040. 10.1080/01431161.2010.484430 (2011).

[29] Pour, A. B. & Hashim, M. The application of ASTER remote sensing data to porphyry copper and epithermal gold deposits. *Ore Geol. Rev.* **44**, 1–9. 10.1016/j.oregeorev.2011.09.009 (2012).

[30] Pour, A. B., Hashim, M. & Marghany, M. Using spectral mapping techniques on short wave infrared bands of ASTER remote sensing data for alteration mineral mapping in SE Iran. *Int. J. Phys. Sci.* **6**(4), 917–929. 10.5897/IJPS10.510 (2011).

[31] Pour, A. B., Hashim, M., Park, Y. & Hong, J. K. Mapping alteration mineral zones and lithological units in Antarctic regions using spectral bands of ASTER remote sensing data. *Geocarto Int.* **33**(12), 1281–1306. 10.1080/10106049.2017.1347207 (2018).

[32] Rajendran, S. et al. Detection of hydrothermal mineralized zones associated with listwaenites in Central Oman using ASTER data. *Ore Geol. Rev.* **53**, 470–488. 10.1016/j.oregeorev.2013.02.008 (2013).

[33] Saed, S. et al. Hydrothermal alteration mapping using ASTER data, Takab-Baneh area, NW Iran: A key for further exploration of polymetal deposits. *Geocarto Int.* **37**(26), 11456–11482. 10.1080/10106049.2022.2059110 (2022).

[34] Kamh, S., Khalil, H., Mousa, G., Abdeen, M. & Ghobara, O. Utilizing remote sensing and lithostratigraphy for iron and clay minerals mapping in the North of Aswan Area, Egypt. *Delta J. Sci.* **44**(2), 1–16 (2022).

[35] El Bastawesy, M., Gabr, S. & White, K. Hydrology and geomorphology of the upper White Nile lakes and their relevance for water resources management in the Nile basin. *Hydrol. Process.* **27**(2), 196–205 (2013).

[36] Attia, M. Topography, geology, and iron ore deposits of the district east of Aswan. Geology Survey, Cairo, Egypt. Earth Sciences Library (Branner) (1955).

[37] El Sharkawi, M. A., El Aref, M. M. & Mesaed, A. A. Stratigraphic setting and paleoenvironment of the Coniacian-Santonian ironstones of Aswan, South Egypt. *Geol. Soc. Egypt. Spec. Publ.* **2**, 243–278 (1996).

[38] Salama, W. Paleoenvironmental significance of aluminum phosphate-sulfate minerals in the upper Cretaceous ooidal ironstones, E-NE Aswan area, southern Egypt. *Int. J. Earth Sci.* **103**(6), 1621–1639. 10.1007/s00531-014-1027-4 (2014).

[39] Nakhla, F. M. & Shehata, M. R. N. Contributions to the mineralogy and geochemistry of some iron-ore deposits in Egypt (UAR). *Miner. Depos.* **2**, 357–371 (1967).

[40] Gomaa, M. M. Heterogeneity in relation to electrical and mineralogical properties of hematitic sandstone samples. *Appl. Water Sci.* **10**, 105. 10.1007/s13201-020-01186-3 (2020).

[41] Shebl, A. & Csámer, Á. Reappraisal of DEMs, Radar and optical datasets in lineaments extraction with emphasis on the spatial context. *Remote Sens. Appl. Soc. Environ.* **24**, 100617. 10.1016/j.rsase.2021.100617 (2021).

[42] Abd El-Wahed, M. A., Kamh, S. Z., Ashmway, M. & Shebl, A. Transpressive Structures in the Ghadir Shear Belt, Eastern Desert, Egypt: Evidence for partitioning of oblique convergence in the Arabian-Nubian Shield during Gondwana Agglutination. *Acta Geol. Sin. English Ed.* **93**, 1614–1646. 10.1111/1755-6724.13882 (2019).

[43] AbdEl-Wahed, M. A., Lebda, E., Ali, A., Kamh, S. & Attia, M. The structural geometry and metamorphic evolution of the Umm Gheig shear belt, Central Eastern Desert, Egypt: Implications for exhumation of Sibai Core Complex during oblique transpression. *Arab. J. Geosci.* **12**, 764. 10.1007/s12517-019-4760-y (2019).

[44] Khan, S. D., Mahmood, K. & Casey, J. F. Mapping of Muslim Bagh ophiolite complex (Pakistan) using new remote sensing, and field data. *J. Asian Earth Sci.* **30**(2), 333–343. 10.1016/j.jseaes.2006.11.001 (2007).

[45] Hashim, M., Pournamdary, M. & Pour, A. B. Processing and interpretation of advanced space-borne thermal emission and reflection radiometer (ASTER) data for lithological mapping in ophiolite complex. *Int. J. Phys. Sci.* **6**(28), 6410–6421. 10.5897/ijps11.417 (2011).

[46] Abaya, H. H., Legesseb, D., Suryabhagavan, K. V. & Atnafub, B. Mapping of ferric (Fe3+) and ferrous (Fe2+) iron oxides distribution using ASTER and Landsat 8 OLI data in Negash Lateritic iron deposit Northern Ethiopia. *Geol. Ecol. Landsc.*10.1080/24749508.2022.2130556 (2022).

[47] Ghoneim, S. M., Yehia, M. A., Salem, S. M. & Ali, H. F. Integrating remote sensing data, GIS analysis and feld studies for mapping alteration zones at Wadi Saqia area, central Eastern Desert Egypt. *Egypt. J. Remote Sens. Space. Sci.* **25**(1), 323–336 (2022).

[48] Boardman, J. W. & Kruse, F. A. Automated spectral analysis: A geological example using AVIRIS data, North Grapevine Mountains, Nevada. In: *ERIM, Ed., Proceeding 10th Thematic Conference on Geological Remote Sensing, San Antonio*, 9–12 May 1994, 407–418 (1994).

[49] Abd El-Wahed, M. A. et al. Multisensor satellite data and field studies for unravelling the structural evolution and gold metallogeny of the Gerf Ophiolitic Nappe, Eastern Desert, Egypt. *Remote Sens.* **15**, 1974. 10.3390/rs15081974 (2023).

[50] Kruse, F. A. et al. The spectral image processing system (SIPS)-interactive visualization and analysis of imaging spectrometer data. *Remote Sens. Environ.* **44**(2–3), 145–163 (1993).

[51] Abedi, M., Norouzi, G.-H. & Bahroudi, A. Support vector machine for multi-classification of mineral prospectivity areas. *Comput. Geosci.* **46**, 272–283. 10.1016/j.cageo.2011.12.014 (2012).

[52] Ducart, D. F., Silva, A. M., Toledo, C. L. B. & Assis, L. M. D. Mapping iron oxides with Landsat-8/OLI and EO1/Hyperion imagery from the Serra Norte iron deposits in the Carajas Mineral Province, Brazil. *Braz. J. Geol.* **46**(3), 331–349 (2016).

[53] Cardoso-Fernandes, J., Teodoro, A. C. & Lima, A. Remote sensing data in lithium (Li) exploration: A new approach for the detection of Li-bearing pegmatites. *Int. J. Appl. Earth Observ. Geoinform.* **76**, 10–25. 10.1016/j.jag.2018.11.001 (2019).

[54] Harris, A. T. Spectral mapping tools from the earth sciences applied to spectral microscopy data. *Cytometry A* **69A**(8), 872–879. 10.1002/cyto.a.20309 (2006). 10.1002/cyto.a.20309 16969808

[55] Bailey, W. R., Walsh, J. J. & Manzocchi, T. Fault populations, strain distribution and basement fault reactivation in the East Pennines Coalfield, UK. *J. Struct. Geol.* **27**, 913–928. 10.1016/j.jsg.2004.10.014 (2005).

[56] El Amawy, M. A., Muftah, A. M., Abd El-Wahed, M. A. & Nasar, A. Wrench structural deformation in Ras Al Hilal-Al Athrun area, NE Libya: A new contribution in northern Al Jabal Al Akhdar Belt. *Arab. J. Geosci.* **4**(7–8), 1067–1085 (2011).

[57] Kim, Y.-S., Andrews, J. R. & Sanderson, D. J. Reactivated strike–slip faults: Examples from north Cornwall, UK. *Tectonophysics* **340**, 173–194. 10.1016/S0040-1951(01)00146-9 (2001).

[58] Korneva, I. et al. Structural properties of fractured and faulted Cretaceous platform carbonates, Murge Plateau (southern Italy). *Mar. Pet. Geol.* **57**, 312–326. 10.1016/j.marpetgeo.2014.05.004 (2014).

[59] Nixon, C. W. et al. Fault interactions and reactivation within a normal-fault network at Milne Point, Alaska. *AAPG Bull.* **98**(10), 2081–2107. 10.1306/04301413177 (2014).

[60] Meshref, W. M. Tectonic framework. In *In the Geology of Egypt* (ed. Said, R.) 113–155 (A. A. Balkema, 1990).

[61] Khedr, E. S., Youssef, A.A., Abu Elmagd, K. & Khozyem, H. M. Tectono-Stratigraphic subdivision of the clastic sequence of Aswan Area, Southern Egypt. In: *Fifth International Conference on Geology of Tethys Realm, South Valley University, Qena, Egypt*, 197–216 (2010).

[62] Mahmoud, S. A. & Williams-Jones, A. E. The rare metal deposits of the El Garra El Hamra syenites, South Western Desert, Egypt. *Ore Geol. Rev.* **101**, 609–628 (2018).

[63] Mostafa, A., El-Barkooky, A. & Hammed, M. Structural geometry and tectonic evolution of an Early Cretaceous rift crossing the Nile Valley in Upper Egypt. *Mar. Pet. Geol.* **153**, 106289 (2023).

[64] Guiraud, R., Issawi, B. & Bosworth, W. Phanerozic history of Egypt and surrounding areas. In *Peri-Tethys Memoir 6: Peri-Tethyan Rift/Wrench Basins and Passive Margins* (eds Ziegler, P. A. et al.) 469–509 (Memoires du Museum National d Histoire Naturelle, 2001).

[65] Abd El-Wahed, M. A. & Thabet, E. Strain geometry, microstructure and metamorphism in the dextral transpersional Mubarak shear belt, Central Eastern Desert, Egypt. *Geotectonics* **51**(4), 438–462 (2017).

[66] Abd El-Wahed, M. A. & Attia, M. Genesis of the gneissic core complexes in the Arabian-Nubian Shield and its tectonic implications: A regional overview. *J. Asian Earth Sci.* **236**, 105337. 10.1016/j.jseaes.2022.105337 (2022).

[67] Abd El-Wahed, M. A. & Attia, M. Structural and tectonic evolution of suture-related belts and post-accretionary systems in the Arabian-Nubian Shield. *Geol. J.* **1**, 1–34. 10.1002/gj.4693 (2023).

[68] Abd El-Wahed, M. A., Ashmawy, M. H. & Tawfik, H. A. Structural setting of Cretaceous pull-apart basins and Miocene extensional folds in Quseir-Umm Gheig region, northwestern Red Sea, Egypt. *Lithosphere* **2**, 13–32. 10.1130/L27.1 (2010).

[69] Bosworth, W. Geological evolution of the Red Sea: Historical background, review, and synthesis. In *The Red Sea: Springer Earth System Sciences* (eds Rasul, N. & Stewart, I.) 45–78 (Springer, 2015). 10.1007/978-3-662-45201-1_3.

[70] Le Pichon, X. & Francheteau, J. A plate tectonic analysis of the Red Sea-Gulf of Aden area. *Tectonophysics* **46**, 369–406 (1978).

[71] Watchorn, F., Nichols, G. J. & Bosence, D. W. J. Rift-related sedimentation and stratigraphy, southern Yemen (Gulf of Aden). In *Sedimentation and Tectonics of Rift Basins: Red Sea-Gulf of Aden* (eds Purser, B. H. & Bosence, D. W. J.) 165–189 (Chapman and Hall, 1998).

[72] Vail, J. R. Outline of the geology and mineral deposits of the Democratic Republic of the Sudan and adjacent areas. Overseas geology and mineral resources 49 (include the Geologic Map of Sudan, scale 1:2:000,000, north and south sheets 1203A and 1203B) (1978).

[73] Guiraud, R. & Bosworth, W. Phanerozoic geodynamic evolution of northeastern Africa and the northwestern Arabian platform. *Tectonophysics* **315**, 73–108 (1999).

[74] Afifi, A. S., Moustafa, A. R. & Helmy, H. M. Rift domains and structural framework of the northwestern Red Sea basin. *Egypt. Int. J. Earth Sci. (Geol. Rundsch.)* **112**, 2049–2064. 10.1007/s00531-023-02340-3 (2023).

[75] Moustafa, A. R. Mesozoic-cenozoic deformation history of Egypt. In *The Geology of Egypt Regional Geology Reviews* (eds Hamimi, Z. et al.) 253–294 (Springer, 2020).

[76] Burke, K. The African plate. *S. Afr. J. Geol.* **99**, 341–409 (1996).

[77] Abdel-Fattah, M. I., Shendi, E. H., Kaiser, M. F. & Abuzied, S. M. Unveiling geothermal potential sites along Gulf of Suez (Egypt) using an integrated geoscience approach. *Terra Nova* **33**, 306–319 (2020).

[78] Faris, M. I. & Abu Zeid, M. M. Age, origin and mining geology of the Aswan Iron ore. *Ain Shams Sci. Bull.* **7**, 85–103 (1961).

[79] Tosson, S. A volcanogenic origin of Aswan iron ore deposits. *Bull. Sac. Sci. Alexanderia Univ. Egypt* **5**, 137–148 (1961).

[80] Schwarz, T. & Germann, K. Ferricretes as a source of continental oolitic ironstones in northern Sudan. *Chem. Geol.* **107**(3–4), 259–265 (1993).

[81] Germann, K., Mocke, A., Doering, T. & Fischer, K. Late Cretaceous laterite-derived sedimentary deposits (oolitic ironstone, kaolins, bauxites) in upper Egypt. *Berliner Geowiss. Abh.* **75**(3), 727–758 (1987).

[82] Baioumy, H., Omran, M. & Fabritius, T. Mineralogy, geochemistry and the origin of high-phosphorus oolitic iron ores of Aswan, Egypt. *Ore Geol. Rev.* **80**, 185–199 (2017).

[83] Youssef, M. A. *Integrated Geophysical Prospecting of the Iron Ore Deposits at East of Aswan, Upper Egypt*. Ph. D. Thesis, Ain Shams University, Egypt (2018).

[84] Abouzeid, A. M. & Khalid, A. M. Mineral industry in Egypt-part I: Metallic mineral commodities. *Nat. Resourc.* **2011**(2), 35–53. 10.4236/nr.2011.21006 (2011).

